# Homogeneous Elongation of N‐Doped CNTs over Nano‐Fibrillated Hollow‐Carbon‐Nanofiber: Mass and Charge Balance in Asymmetric Supercapacitors Is No Longer Problematic

**DOI:** 10.1002/advs.202200650

**Published:** 2022-05-14

**Authors:** Taewoo Kim, Subhangi Subedi, Bipeen Dahal, Kisan Chhetri, Tanka Mukhiya, Alagan Muthurasu, Jagadis Gautam, Prakash Chandra Lohani, Debendra Acharya, Ishwor Pathak, Su‐Hyeong Chae, Tae Hoon Ko, Hak Yong Kim

**Affiliations:** ^1^ Department of Nano Convergence Engineering Jeonbuk National University Jeonju 54896 Republic of Korea; ^2^ Department of Chemistry Trichandra Multiple Campus Tribhuvan University Kathmandu 44605 Nepal; ^3^ Central Department of Chemistry Tribhuvan University Kathmandu 44618 Nepal; ^4^ Department of Chemistry Bhaktapur Multiple Campus Tribhuvan University Kathmandu 44800 Nepal; ^5^ School of Materials Science and Engineering Kumoh National Institute of Technology 61 Daehak‐ro Gumi‐si Gyeongsangbuk‐do 39177 Republic of Korea; ^6^ Department of Chemistry Amrit Campus Tribhuvan University Kathmandu 44600 Nepal; ^7^ Department of Organic Materials and Fiber Engineering Jeonnbuk National University Jeonju 561–756 Republic of Korea

**Keywords:** double layer anodes, electrospinning, mass balancing, metal–organic frameworks, nano‐fibrillated hollow carbon nanofibers (CNFs), N‐doped carbon nanotubes (CNTs), supercapacitors

## Abstract

The hurdle of fabricating asymmetric supercapacitor (ASC) devices using a faradic cathode and a double layer anode is challenging due to the required large amount of active mass of anodic material compared to that of the cathodic material during mass balancing due to the large difference in capacitance values of the two electrodes. Here, the problem is addressed by engineering a negative electrode that furnishes an ultrahigh capacitance. An in situ developed metal–organic framework (MOF)‐based thermal treatment is adopted to grow highly porous N‐doped carbon nanotubes (CNTs) containing submerged Co nanoparticles over nano‐fibrillated electrospun hollow carbon nanofibers (HCNFs). The optimized CNT@HCNF‐1.5 furnishes an ultrahigh capacitance approaching 712 F g^–1^ with excellent rate capability. The capacitance reported from this work is the highest for any carbonaceous material reported to date. The CNT@HCNF‐1.5 is further used to fabricate symmetric supercapacitors (SSCs), as well as ASC devices. Remarkably, both the SSC and ASC devices furnish incredible performances in all aspects of SCs, such as a high energy density, long cycle life, and high rate capability, displaying decent practical applicability. The energy density of the SSC device reaches as high as 20.13 W h kg^–1^, whereas that of ASC approaches 87.5 W h kg^–1^.

## Graphical abstract

An in situ developed metal–organic framework‐based thermal treatment technique is adopted to prepare porous N‐doped carbon nanotubes containing firmly submerged Co‐nanoparticles over nano‐fibrillated electrospun hollow carbon nanofibers. When the optimized CNT@HCNF‐1.5 is applied as a negative electrode, the problem related to mass balancing during fabrication of asymmetric supercapacitor can be addressed satisfactorily that will open new possibilities for the future works.

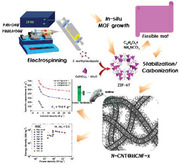

## 1 Introduction

Supercapacitors (SCs) are considered ideal devices for short‐term energy storage because they have the ability to quickly charge and discharge electrical energy, and they possess an ultrahigh power density with long‐term cyclic stability.^[^ ^1^ ^]^ However, SCs have a major limitation in that they do not have a high capacitance, and hence a high energy density.^[^ ^1^ ^]^ The simultaneous improvement of three major issues of SCs, namely, high capacitance, high energy density, and high cyclic stability, is mainly limited by the low specific capacitance of carbon‐based negative electrode materials.^[^ ^2^ ^]^ Having thoroughly investigated the most recent articles published in reliable sources, it has been determined that the specific capacitances of common carbonaceous negative electrodes are mostly in the range of 100—500 F g^–1^, which is ≈10–20% of the capacitances of positive electrodes.^[^ ^3^, ^4^, ^5^, ^6^ ^]^ When we wish to fabricate hybrid SCs that provide a higher potential window and a higher capacitance, and hence a higher energy density, three‐ to fivefold or even more negative electrode material is required to bridge the capacitance gap between the positive and negative electrode materials, which may limit the energy density of the device. Therefore, it is not practical to balance mass and charge during hybrid SC device fabrication.

Carbon nanotubes (CNTs) are an astonishing invention for science and technology, with open tubular structures and enriched chirality that can be used in energy storage systems due to their high electronic conductivity, large surface area, superior electrochemical and mechanical stability, and their light weight.^[^ ^7^, ^8^, ^9^ ^]^ However, proper engineering of CNTs with a low cost and facile technologies is still a nuisance task. Various synthetic techniques, such as chemical vapor deposition (CVD), arc discharge, laser ablation, and solid‐state cutting, have been adopted by many researchers.^[^ ^10^, ^11^, ^12^, ^13^ ^]^ Nevertheless, these techniques suffer from high production costs, a lack of controlled assembly, and confinement of electrochemically active species within the carbon framework.^[^ ^14^, ^15^ ^]^

Here, we fabricated highly porous N‐doped CNTs (N‐CNTs) stretched out on an N‐doped carbon nanofibrous material, leading to a surprisingly high capacitance. A noble chemical treatment technique was adopted to make highly flexible CNT‐grown carbon nanofiber mats, which can be used as free‐standing electrodes for supercapacitors. To improve the capacitance and energy density, herein, a promising method has been suggested that has no adverse effects on carbon nanofiber (CNF)‐based carbon materials without adding substantial pseudocapacitive groups. It is worth mentioning here that enhancing the pseudocapacitance by introducing too many functional groups obviously decreases the stability (both electrochemical and mechanical) and conductivity of the material.^[^ ^15^ ^]^ The synthetic method includes increasing the effective surface area by optimizing the pore structure and distribution with the addition of an optimum amount/number of hetero‐elements and metal nanoparticles (NPs) within elongated CNTs and base CNFs as electron‐rich centers for the absorption of cations.

**Figure 1:**
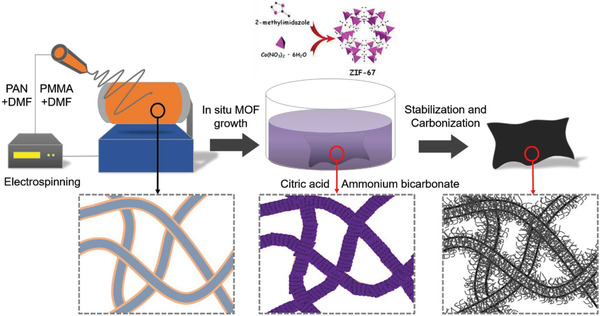
Schematic illustration of the fabrication technique of the CNT@HCNF‐x electrode.

A facile and inexpensive in situ‐grown metal‐organic framework (MOF)‐based thermal treatment technique was adopted without using any complex or expensive instruments, additional precursors or reducing gases, as used previously in existing synthesis methods (**Figure** **1**). Nanometer‐sized cobalt and 2‐methylimidazole‐based MOFs (ZIF‐67) were uniformly synthesized over electrospun polyacrylonitrile (PAN)‐ and polymethylmethacrylate (PMMA)‐based sheath‐core nanofibers, facilitating the growth and proper orchestration of N‐CNTs on hollow CNFs (HCNFs) after an optimum heat treatment. Most importantly, at optimum precursor ratios and heat treatment temperatures, a uniform bush of highly porous N‐CNTs was successfully grown over the nano‐fibrillated and hollow CNFs, leaving single and aggregated Co‐NPs firmly submerged into the N‐CNTs with high aspect ratios (up to 3 µm long and 5–20 nm in diameter). The as‐prepared samples were then tested as SC electrodes in both symmetric and asymmetric fashioned devices. The exceptional capacitive performance of the optimized sample safeguards the most problematic issue of mass and charge balancing between positive and negative electrodes in asymmetric SCs and opens the door to fabricate and commercialize flexible and lightweight extraordinary performer symmetric SC devices.

## 2 Results and Discussion

### 2.1 Physico‐Chemical Characterizations

**Figure 2:**
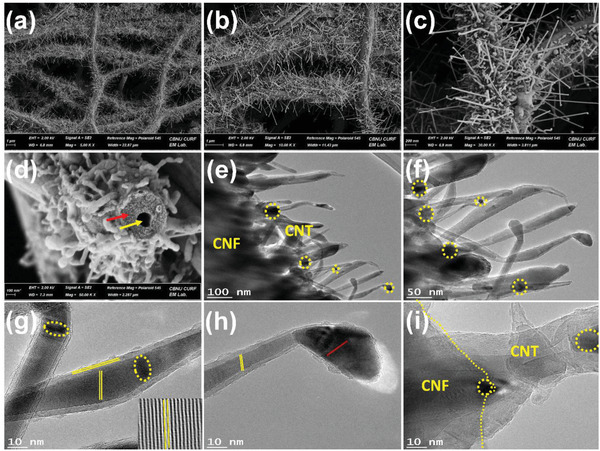
a–c) FE‐SEM images of CNT@HCNF‐1.5 at different magnifications, d) cross‐section showing hollow fibers and nanofibrils, e,f) TEM images, g,h) HR‐TEM showing lattice fringes and connections between CNFs and CNTs (i).

The surface morphology of the CNT@HCNF‐1.5 flexible nanofiber membranes presented in **Figure** **2**a–c shows the dense growth and excellent coverage of nano‐fibrillated HCNFs by N‐CNTs with high aspect ratios (up to 3 µm long and 5–20 nm in diameter). The cross‐section morphology shows the core of hollow CNFs (indicated by the yellow cursor) and the sheath of nanofibrils (indicated by the red cursor). Furthermore, the microstructure of the sample was studied using TEM images, which show a uniform bush of highly porous N‐CNT HCNFs leaving single and aggregated Co‐NPs firmly submerged into the N‐CNTs (Figure 2e,f). The portion highlighted by red circles in the figure shows the aggregation of Co‐NPs, while single Co‐NPs can be observed throughout the sample, which can also be observed in the HR‐TEM images (Figure 2g–i). The fine lattice fringes displayed in HR‐TEM indicate the crystallinity and graphitic nature of the material.^[^ ^16^ ^]^ The distinct crystalline planes with lattice fringes of 0.35 and 0.21 nm correspond to the (002) plane of graphitic carbon and the (111) plane of metallic Co, respectively. The same result with three distinct rings is noticed from the SAED patterns taken at the area containing both Co‐NPs and graphitic carbon (Figure S1, Supporting Information). Fringes corresponding to the crystalline plane of graphitic carbon are observed in both N‐CNTs and core HCNFs. Some Co‐NPs with corresponding lattice fringes are also observed in the joint between CNTs and HCNFs (Figure 2i). Furthermore, EDX elemental mapping of the sample confirms the uniform distribution of Co along with C, O, and N, indicating an intact structure and uniform porosity throughout the sample (Figure S2, Supporting Information). The EDX spectrum analysis and weight percent of different elements are in ideal agreement with the results obtained from the XPS analysis (Figure S2, Supporting Information). The FE‐SEM images of CNT@HCNF‐1, CNT@HCNF‐2, and HCNF (Figures S3 and S4, Supporting Information) and the respective EDX spectra and elemental mapping (Figures S5 and S6, Supporting Information) of CNT@HCNF‐1 and CNT@HCNF‐2 are presented in the Supporting Information. Low populated N‐CNTs with low aspect ratios are observed in CNT@HCNF‐1, whereas CNT@HCNF‐2 displays the aggregation of N‐CNTs. However, the distribution of elements is uniform throughout the sample in all cases. The morphology of all the samples before heat treatment is presented in Figure S7 (Supporting Information), showing the growth pattern of ZIF‐67 and its coverage over the core polymer nanofibers as a ZIF‐67@PAN/PMMA moiety.

**Figure 3:**
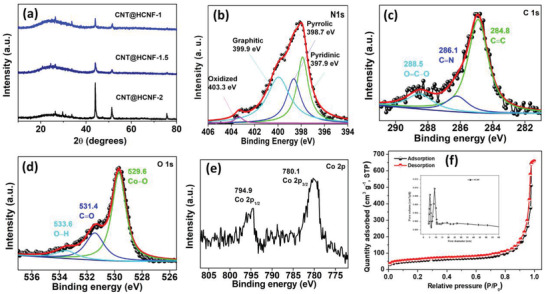
a) XRD patterns of different samples. High‐resolution XPS spectrum of CNT@HCNF‐1.5 deconvoluted for b) N 1s, c) C 1s, d) O 1s, e) Co 2p, and f) BET adsorption–desorption isotherms for CNT@HCNF‐1.5 (inset: pore‐size distribution curve).

**Figure** **3**a displays the typical XRD patterns of the prepared CNT@HCNF‐1, CNT@HCNF‐1.5, and CNT@HCNF‐2. The XRD patterns of all the samples approximately match the standard reference peaks of carbon and Co‐NPs. The broad peaks at 24.8 correspond to the (002) lattice plane of graphitic carbon. The peaks at 44.2, 51.4, and 76.8 represent the (111), (200), and (220) lattice planes of metallic Co‐NPs, respectively. Slight shifts in the peak positions and the presence of a few minor peaks with a low intensity are the results of composite formation, few bonds between Co and O, and the defects created by acid washing. The Raman spectrum of CNT@HCNF‐1.5, presented in Figure S8 (Supporting Information), provides information about the defects and graphitization of the material. The ratio of the intensities of the graphitic peak observed at 1570 cm^–1^ and that of the peak representing defects observed at 1345 cm^–1^ (*I*_G_/*I*_D_) is ≈1.11, indicating that the high degree of graphitization of carbon results in the high conductivity of the material.^[^ ^17^, ^18^ ^]^ The chemical states and bonding in CNT@HCNF‐1.5 among C, O, N, and Co were examined by XPS. The low‐resolution survey spectrum (Figure S9, Supporting Information) provides the percentage of all the elements present in the samples, which are comparable to the results obtained from EDX. The deconvoluted high‐resolution XPS spectra of N, C, and O with their respective peak positions are presented in Figure 3b–d. The types of N and possible chemical states of N, C, and O are perfectly matched with many standard references.^[^ ^19^, ^20^ ^]^ The existence of strong C═C, C═N, O═C—O, and C═O bonds and the presence of pyridinic, pyrrolic, graphitic, and oxidized nitrogen confirm the graphitization on CNT@HCNF‐1.5 and nitrogen doping in N‐CNTs. Similarly, the existence of Co—O bonds provides proof for the distribution of Co‐NPs in the as‐prepared samples. However, the spectrum of Co 2p could not be perfectly deconvoluted into different chemical states, indicating the presence of metallic Co in the zero valance state with strong peaks at 280.1 eV for Co 2p_3/2_ and at 794.9 eV for Co 2p_1/2_ (Figure 3e). The results obtained from the N_2_ adsorption–desorption experiment reveal that CNT@HCNF‐1.5 possesses a BET surface area of 812.6 m^2^ g^–1^ with a total pore volume of 1.18 cm^3^ g^–1^ and an average pore diameter of 9.8 nm, which are ideal conditions for hydrated ion transportation within the material, ensuring more adequate electrochemical reactions (Figure 3f).^[^ ^21^ ^]^ This is one of the major reasons why CNT@HCNF‐1.5 gives an exceptional electrochemical performance attributed to the high capacitance and energy density. The adsorption isotherms and pore size distribution of the counter control samples are provided in Figure S10 (Supporting Information). The BET texture properties of the prepared samples are organized in Table S1 (Supporting Information). The large surface area is due to the highly porous nature of N‐CNTs and nano‐fibrillated HCNFs, which obviously increases the hydrophilicity of the material. It has been reported that lone pairs of electrons present in pyridinic N also increase the hydrophilic characteristics of carbon materials.^[^ ^22^ ^]^ Furthermore, the inductive effect generated by sigma electrons operates on nonadjacent atoms, which further increases the hydrophilicity of the carbon materials. The contact angle measurements of the samples in aqueous 2 m KOH are given in Figure S11 (Supporting Information).

### 2.2 Electrochemical Performance

The fabrication process and morphology of electrode materials play a vital role in the enhanced performance of SCs. The capacitance of an SC is highly dependent on the surface area of the electrode, porosity, electrochemically active sites, and selection of electrolytes. For materials that store energy using an electrochemical double layer capacitance (EDLC) mechanism, charge separation at the electrode–electrolyte interface, transportation of ions between two electrodes, and diffusion of ions across the electrolyte are fundamental issues. The fabricated electrode CNT@HCNF‐1.5 satisfied all these circumstances, and hence, delivered an ultrahigh capacitance. N‐CNTs grown over nano‐fibrillated HCNFs have a high mesoporosity and a high surface area with N as a heteroatom, which modifies the conduction band with a modified electronic structure and turns carbon into an electrochemically active species.^[^ ^23^ ^]^ It has been reported that lone pairs of electrons present in pyridinic N increase the hydrophilic characteristics of carbon materials.^[^ ^22^ ^]^ Furthermore, the inductive effect generated by sigma electrons operates on nonadjacent atoms, which further increases the hydrophilicity of carbon materials.^[^ ^24^ ^]^ Additionally, the nano‐fibrillated and hollow CNFs with single and aggregated Co‐NPs firmly submerged into the N‐CNTs provide much higher, continuous, and fast electronic conductivity (Table S2, Supporting Information). Consequently, N doping, the presence of oxygen functional groups, and the existence of Co–O bonds (as observed in the XPS study) generate some pseudocapacitance in the carbon materials apart from the majority of EDLCs.

The electrochemical properties of the CNT@HCNF‐1.5 and other counter control samples were evaluated using CV, GCD, and EIS techniques, first in a three electrode configuration in 2 m KOH as an electrolyte and later in a two electrode system by fabricating both symmetric SC (SSC) and asymmetric SC (ASC) devices using PVA/KOH gel as an electrolyte. The flexible CNT@HCNF‐1.5 was directly used as a free‐standing electrode in a three‐electrode system and in a two‐electrode symmetric SC device. In the asymmetric SC device, it was coated over nickel foam by making slurry using a polymer binder and conductive additive in a suitable volume of organic solvent. The most widely studied Co_3_O_4_ was used as a positive electrode, particularly to illustrate the convenience of mass balance during the fabrication of ASCs.

**Figure 4:**
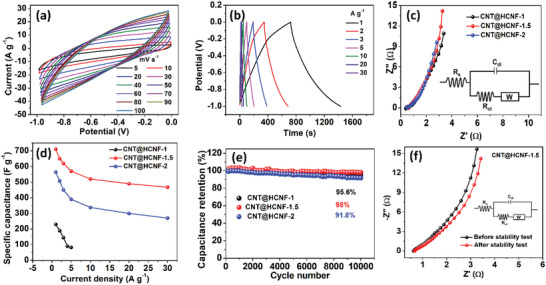
Electrochemical characterizations of CNT@HCNF‐1.5. a) CV curves at different scan rates, b) GCD curves at different current densities, c) Nyquist impedance plots for different samples (inset: equivalent circuit diagram used to fit Nyquist plots using ZSimpWin software), d) specific capacitances with respect to current densities for different samples, e) plot showing the cyclic stability of different samples, f) comparison of Nyquist plots before and after stability test (inset: equivalent circuit diagram).

The CV studies reveal that CNT@HCNF‐1.5 demonstrated a typical capacitive behavior with nearly rectangular CV curves, not including obvious redox peaks (**Figure** **4**a). The deviation from the rectangular shape is due to the N‐dopant and other oxygen‐containing functional groups. This indicates that the charge storage mechanism is EDLC‐dominant along with some pseudocapacitance. The unchanged shape of the CV curves up to 100 mV s^–1^ proved the rapid ion diffusion rates. Likewise, the symmetric GCD curves of CNT@HCNF‐1.5 approaching a triangular shape from 1 to 30 A g^–1^ further conform to the capacitive behavior of the electrode (Figure 4b). Based on the GCD curves, the specific capacitance (*C*_s_) values were calculated as a function of the current density. The CNT@HCNF‐1.5 furnished an ultrahigh *C*_s_ value approaching 712 F g^–1^ at a current density of 1 A g^–1^ and maintained 67.7%, even at 30 A g^–1^ (Figure 4d). The *C*_s_ value of CNT@HCNF‐1.5 reported from this work is the highest for any carbonaceous material reported to date (Table S6, Supporting Information).^[^ ^8^, ^25^, ^26^, ^27^, ^28^, ^29^, ^30^, ^31^, ^32^, ^33^, ^34^ ^]^ This is one of the keys and astonishing results of this study; hence, the title “mass and charge balancing in ASCs is no longer problematic.” Due to the high capacitance value provided by carbonaceous negative electrodes with the majority of EDLCs, it was possible to fabricate an ASC device that used the mass of positive and negative electrode materials at a ratio of 1:1 even though the positive electrode (the most widely studied pseudocapacitive/battery‐type material, Co_3_O_4_) furnished a very high capacitance of 1790 F g^–1^. In Figure 4d, the *C*_s_ values of CNT@HCNF‐1, CNT@HCNF‐1.5, and CNT@HCNF‐2 are also compared at different current densities. The CV and GCD curves of CNT@HCNF‐1 and CNT@HCNF‐2 are presented in Figures S12 and S13 (Supporting Information). Furthermore, each CNT@HCNF‐1, CNT@HCNF‐1.5, and CNT@HCNF‐2 electrode was tested to check its cyclic stability in 2 m KOH for 10 000 continuous charge–discharge cycles at a high current density of 20 A g^–1^ (Figure 4e). The results revealed that all three electrodes preserved >90% of their initial capacitance, with CNT@HCNF‐1.5 being superior with a retention of 98% of its initial capacitance. This excellent galvanostatic stability is attributed to the formation of an electrical double layer as the dominant charge storage mechanism, without deformation of the electrode morphology (Figure S16, Supporting Information). Furthermore, strong covalent bonds among C, N, O, and Co prevent structural deformation, even though they contribute to pseudocapacitance via redox reactions. The post‐stability‐test analysis results of CNT@HCNF‐1.5 are presented Figures S17–S19 (Supporting Information).

Furthermore, the Nyquist impedance plots obtained from EIS for the CNT@HCNF‐1.5 and their control counters CNT@HCNF‐1 and CNT@HCNF‐2, presented in Figure 4c, did not display any well‐defined arc or semicircle in the high‐frequency region, demonstrating a low resistance at the bulk of the solution. This indicates that there is no noncharging regime, i.e., the electrodes can be charged at any range of frequencies due to the high surface area with a suitable pore size and high conductivity of the electrodes provided by N‐CNTs and homogenously embedded Co‐NPs.^[^ ^35^ ^]^ A nearly vertical line appeared in the low‐frequency region with a negligible semicircle diameter, proving the synchronous charging and discharging and low voltage drop (and hence the low equivalent series resistance, ESR), which is proportional to the capacitance and high electronic conductivity of the electrodes.^[^ ^36^, ^37^, ^38^ ^]^ A comparison of the Nyquist impedance parameters of the CNT@HCNF‐1, CNT@HCNF‐1.5, and CNT@HCNF‐2 electrodes obtained after fitting by ZSimpWin software is presented in Table S3 (Supporting Information). A comparison of the Nyquist impedance plots of the CNT@HCNF‐1.5 electrodes before and after the stability tests, presented in Figure 4f, displayed almost similar curves with negligible increases in each impedance parameter: intrinsic resistance (*R*_s_), Warburg diffusion resistance (*R*_w_), and charge transfer resistance (*R*_ct_). The low values of *R*_s_, *R*_w_, and *R_ct_* suggest a low ESR and a low voltage drop. It also suggests fast ion transport/diffusion with a high conductivity of the active materials.

The storage mechanism of an EDLC type electrode material is electrostatic adsorption, known as capacitive non‐Faradaic charge storage. However, for the materials based on Faradic charge storage behavior, the term capacity is mostly used in place of capacitance. In case of CNT@HCNF‐1.5 electrode the charge storage mechanism is dominated by capacitive non‐Faradic type, the term capacitance has been used to evaluate the capacitive performance throughout the manuscript.^[^ ^39^ ^]^ The storage mechanism of CNT@HCNF‐1.5 major electrode has been studied using the relation *i* = *av* ^b^. To confirm the charge storage kinetics of electrode, the logarithm relationship between the peak current density (*i*) and scan rate (*v*) is used to distinguish whether the electrochemical process is diffusion‐controlled or surface‐controlled.

(1) $$i=av^{b}$$

(2) log(i)=blog(v)+log(a)

where a and b are the constants. The value of b is equal to 0.5 indicates diffusion‐controlled electrochemical process due to the Faradaic intercalation reaction and a b‐value of 1.0 demonstrates an entirely capacitive non‐Faradic behavior due to surface capacitive effect. For CNT@HCNF‐1.5, the calculated b value is closer to 1.0 (b = 0.952) indicating the dominance of capacitive non‐Faradic type storage mechanism (Figure S14, Supporting Information). From calculations, the capacitive non‐Faradic contribution was ≈80% at scan rate of 5 mV s^–1^ which increased to ≈93% at 100 mV s^–1^. Further, the coulombic efficiencies of CNT@HCNF‐1.5 and fabricated ASC device are provided in the Figure S15a,b (Supporting Information).

**Figure 5:**
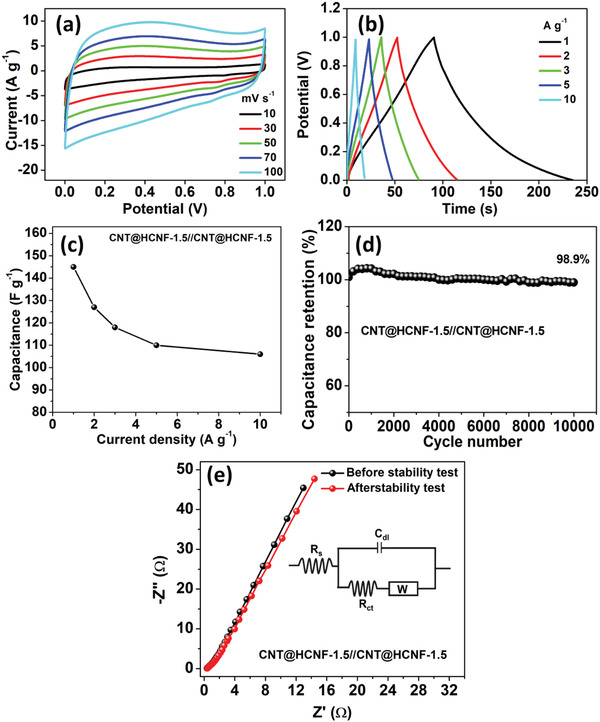
Electrochemical characterizations of the CNT@HCNF‐1.5//CNT@HCNF‐1.5 SSC device. a) CV curves, b) GCD curves, c) plot showing variations in the specific capacitance with current densities, d) plot showing the cyclic stability of SSC device, e) comparison of Nyquist plots before and after the stability test (inset: equivalent circuit diagram).

**Figure 6:**
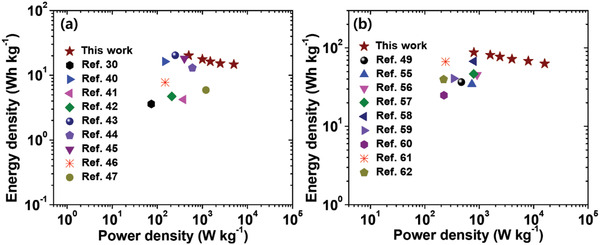
Ragone plots for the a) CNT@HCNF‐1.5//CNT@HCNF‐1.5 SSC device and b) Co_3_O_4_@NF//CNT@HCNF‐1.5 ASC device.

To probe the practical application of the CNT@HCNF‐1.5 electrode, first a flexible all‐solid‐state SSC device was assembled in a sandwich configuration with PVA/KOH gel as an electrolyte. The CV curves (**Figure** **5**a) and GCD curves (Figure 5b) of the as‐fabricated flexible CNT@HCNF‐1.5//CNT@HCNF‐1.5 SSC device were examined at different scan rates (from 10 to 100 mV s^–1^) and different current densities (from 1 to 10 A g^–1^). Approximately rectangular CV curves and almost triangular GCD curves suggest the capacitive behavior of the SSC device. The *C*_s_ value of the cell reached 145 F g^–1^ at a current density of 1 A g^–1^, which maintained an ultrahigh rate capability of 73.1% at 10 A g^–1^ (Figure 5c). Notably, the SSC device maintained a remarkable cyclic stability with a 98.9% capacitance retention, even after 10 000 continuous charge–discharge cycles (Figure 5d). Furthermore, to examine the high‐rate performance of the CNT@HCNF‐1.5//CNT@HCNF‐1.5 SSC device from the perspective of electrode–electrolyte interface behavior, ion diffusion and bulk properties of the gel electrolyte, EIS was performed between the 0.01 Hz to 100 kHz frequency range. The obtained Nyquist impedance plots (Figure 5e) showed that the as‐fabricated SSC device initially suffers from low *R*_s_, *R*_w_, and *R*_ct_, indicating a low ESR (0.29 Ω), which marginally increases to 0.37 Ω after the stability test, suggesting fast ion diffusion, a low voltage drop during the GCD test, and the extraordinary cyclic stability of the SSC device (Table S4, Supporting Information). The impedance parameters were obtained by fitting the curves using ZSimpWin software. From the calculations, the energy density (*E*) of the SSC device reached as high as 20.13 W h kg^–1^ at 1 A g^–1^, which decreased to 14.7 W h kg^–1^ at 10 A g^–1^ as the corresponding power density (*P*) increased from 499.8 W kg^–1^ at 1 A g^–1^ to 4992 W kg^–1^ at 10 A g^–1^ (**Figure** **6**a; Table S7, Supporting Information)). The *E* and *P* values are superior to those of recently reported carbon material‐based SSC devices.^[^ ^30^, ^40^, ^41^, ^42^, ^43^, ^44^, ^45^, ^46^, ^47^ ^]^ After charging, two flexible SSC devices connected in series could power a 1 W white LED for ≈20 min at 20 A g^–1^, which demonstrated the practical applicability of the device (Figure S20, Supporting Information). To the best of our knowledge, our flexible SSC device is one of the best performers in every aspect of electrochemical parameters, including practical applicability. This opens the door for a new possibility of assembling lightweight and flexible SSC devices for commercialization.

The extraordinary capacitive performance of the CNT@HCNF‐1.5 negative electrode motivated us to assemble an ASC device using the most widely used Co_3_O_4_ positive electrode, particularly to illustrate the convenience in mass balance, which is one of the most annoying steps during device fabrication in an asymmetric fashion. The reason for selecting Co_3_O_4_ as a positive electrode is that it is one of the best pseudocapacitive/battery‐type electroactive materials furnishing a high capacitance value, which has been extensively studied by researchers in this field. The same Co_3_O_4_ nanohairs grown over nickel foam used in our previous studies were utilized as positive electrodes for asymmetric fashioned SC devices.^[^ ^48^, ^49^, ^50^, ^51^ ^]^ Therefore, the detailed characterization and electrochemical properties of the positive electrode are not included in this study. The synthesis technique of the positive electrode is included in the Experimental Section, and its electrochemical performances are presented in Figure S21 (Supporting Information).

Most importantly, the Co_3_O_4_@NF positive electrode furnished a high C_s_ of 1790 F g^–1^ at a current density of 1 A g^–1^. Fabrication of ASC devices using such faradic electrodes (with a high capacitance) has always been challenging, mostly in the case of using double layer carbonaceous negative electrodes due to the high active mass requirement of the negative electrode compared to that of the positive electrode. This hurdle has been addressed by using CNT@HCNF‐1.5 as a negative electrode, as it furnished an ultrahigh capacitance.

**Figure 7:**
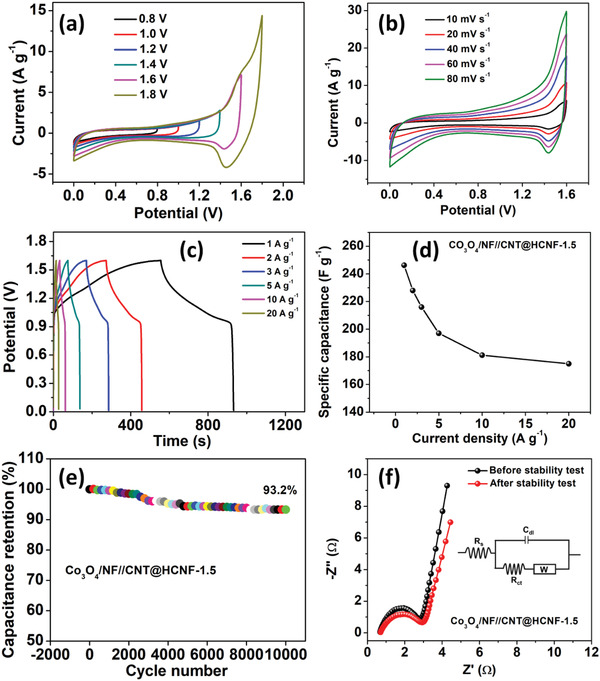
Electrochemical performances of the Co_3_O_4_@NF//CNT@HCNF‐1.5 ASC device. a) CV curves at different working potentials at a scan rate of 20 mV s^–1^, b) CV curves at different scan rates, c) GCD curves at different current densities, d) plot showing the variation in the specific capacitances at different current densities, e) plot showing cyclic stability, f) Nyquist impedance plots before and after the stability test (inset: equivalent circuit diagram).

**Figure** **7**a displays the CV profiles of the as‐fabricated Co_3_O_4_@NF//CNT@HCNF‐1.5 ASC device taken at a scan rate of 20 mV s^–1^ in 2 m KOH as an electrolyte. The consistent overlapping CV curves revealed the good reversibility and capacitive behavior of the device.^[^ ^52^ ^]^ After 1.5 V, a sharp increase in the current was observed due to the oxygen evolution reaction (OER), which may hinder the device working beyond that potential. However, in the GCD test the device worked successfully up to a stable potential window of 1.6 V, offering a reasonable coulombic efficiency. The reason behind the extension of the working potential up to 1.6 V in GCD is the intercalation of K^+^ ions (from electrolyte) into porous electrodes, which forms a passivation layer providing kinetic stability to the device by leveraging the overpotential of OER. The experimental evidence of the K^+^ ions intercalation phenomenon can be illustrated from the XPS studies of the electrode after all electrochemical tests. Figure S17 (Supporting Information) provides the low resolution XPS spectra of CNT@HCNF‐1.5 electrode before and after electrochemical tests. The appearance of K2p peak with 0.47% potassium is the evidence of K^+^ ion intercalation into the electrode that was not visible in the spectrum before electrochemical tests. The formation of passivation layer in supercapacitor electrodes induced by K^+^ ions intercalation is theoretically similar to the spontaneous formation of cathode electrolyte interphase (CEI) in batteries during charge–discharge process at high voltage. The CEI plays important role to stretch the working potential and determining the life of the cell. The Co‐rich negative electrode and the battery type Co_3_O_4_ positive electrode undergo some anionic and cationic redox reactions in presence of K^+^ ions and promote a thin CEI growth during charging and discharging processes. The standard electrode potentials of positive or negative electrodes (*V*_p_ or *V*_n_) correspond to fermi levels of respective electrodes. The energy levels near the fermi level of electrode materials must match with an appropriate lowest unoccupied molecular orbital (LUMO) or highest occupied molecular orbital (HOMO). If the positive electrode has higher *V*_p_ than the HOMO the electrolyte oxidizes to form a passivation layer to mitigate transfer of electron from HOMO of the electrolyte to positive electrode. Similarly, if the negative electrode has lower *V*_n_ than the LUMO, the electrolyte reduces to form passivation layer and hinders the electron transfer from negative electrode to the LUMO of the electrolyte. Therefore, the *V*_p_ and *V*_n_ locate within the theoretical stable window of the electrolyte if there is absence of passivation layer. Nevertheless, when formation of passivation layer takes place at the interface of electrode and electrolyte, it provides the kinetic stability to stretch the working potential of the full cell that exceeds the limit of thermodynamically stable electrolyte window. The extension of the potential window of the fabricated ASC device up to 1.6 V is illustrated voltammetrically in Figure S22 (Supporting Information). The CV curves furnished by the device at different scan rates from 10 to 80 mV s^–1^ are presented in Figure 7b, and the GCD curves taken at different current densities (from 1 to 20 A g^–1^) are given in Figure 7c. Due to parasitic faradic processes CV reveals various HER and OER side‐effects at relatively higher and lower potential ranges, respectively. However, GCD is the constant current experiment that provides actual current distribution below the GCD curves. In our work also CV revealed the considerable inconsistencies due to the pseudocapacitances produced by submerged Co‐nanoparticles and doped heteroatoms in negative electrode and battery type Co_3_O_4_ positive electrode. However, in our results the CE and ESR values are appreciable with low potential drop even at higher current densities as displayed by GCD. The capacitance distribution below 0.3 V in CV results predominates the HER that is not visible in GCD as current is not a variable and capacitance distribution between 0.4 to 1.2 V looks negligible (nonetheless not insignificant in GCD also) in case of CV results too. Even in GCD, the negligible capacitance area (not insignificant) contributes to the energy density calculated by using, *E* = 1/2 CV^2^. In our GCD profiles there seems a large underestimated area even below 0.9 V that contributes effectively to the capacitance.^[^ ^53^ ^]^ The cell capacitance of the device at 1 A g^–1^ reached 246.2 F g^–1^ and retained 71.1% (175 F g^–1^) of its initial capacitance, furnishing the excellent rate capability and demonstrating the good capacitive properties and reversibility of the ASC device (Figure 7d). In addition, the Co_3_O_4_@NF//CNT@HCNF‐1.5 ASC device displays an excellent cyclic stability in 2 m KOH with a 93.2% capacitance retention after 10 000 successive charge–discharge cycles at a high current density of 10 A g^–1^ (Figure 7e). Figure 7f displays the Nyquist plots of as‐fabricated ASC device before and after electrochemical tests. The *R*_s_, *R*_ct_, and *W* hence ESR slightly increases from 2.83 to 3.18 Ω after stability test demonstrating marginal decrease in conductivities and ion diffusion rate.^[^ ^54^ ^]^ The Nyquist impedance parameters of the fabricated ASC device are provided in Table S5 (Supporting Information).

The Ragone plot showing the variation in *E* and *P* is presented in Figure 6b. The fabricated ASC device furnished an *E* of 87.5 W h kg^–1^ with a *P* of 799.9 W kg^–1^ at 1 A g^–1^. The value of E was still maintained at 62.2 W h kg^–1^ with an increase in *P* to 15 999 W kg^–1^ at 20 A g^–1^. The values of *E* and *P* exceed many recently published works using Co_3_O_4_‐based positive and carbon‐based negative electrodes (Table S8, Supporting Information).^[^ ^49^, ^55^, ^56^, ^57^, ^58^, ^59^, ^60^, ^61^, ^62^ ^]^ To check the practical applicability, after charging, two ASC devices were connected in series that could power a 1 W red LED for ≈12 min at 10 A g^–1^ (Figure S23, Supporting Information).

## 3 Conclusions

Highly porous N‐CNTs stretched out on N‐doped nano‐fibrillated and hollow CNFs were successfully synthesized using a noble chemical curing technique, leading to a highly flexible N‐CNT/CNF mat that was used as the free‐standing electrode for supercapacitors and that displayed a surprisingly high capacitance. A facile and inexpensive in situ‐grown metal‐organic framework (MOF)‐based thermal treatment technique has been adopted without using any complex or expensive instruments, additional precursors or reducing gases, as used previously in existing synthesis methods. The as‐prepared CNT@HCNF‐1.5 furnished an ultrahigh *C*_s_ value approaching 712 F g^–1^ at a current density of 1 A g^–1^, the highest for any carbonaceous material reported to date, and maintained 67.7%, even at 30 A g^–1^. Due to the high capacitance value provided by carbonaceous negative electrodes with the majority of EDLCs, it was possible to fabricate an ASC device that used the mass of positive and negative electrode materials at a ratio of 1:1, even though the positive electrode (most widely studied pseudocapacitive/battery‐type material, Co_3_O_4_) furnished a very high *C*_s_ of 1790 F g^–1^. The fabricated ASC device furnished an E of 87.5 W h kg^–1^ with a *P* of 799.9 W kg^–1^. The value of *E* was maintained at 62.2 W h kg^–1^ with an increase in *P* to 15 999 W kg^–1^ in the PVA/KOH gel electrolyte. A flexible all‐solid‐state SSC device was also assembled in a sandwich configuration with a PVA/KOH gel as an electrolyte that furnished a high *E* value of 20.13 W h kg^–1^, which decreased to 14.7 W h kg^–1^, with the corresponding *P* value increasing from 499.8 to 4992 W kg^–1^. Therefore, it is expected that this work will open new possibilities toward engineering the negative electrode materials that solely provide the ultrahigh capacitance to overcome the hurdle of mass balancing in asymmetric supercapacitor with high energy density at the same time. On the other hand, we also successfully fabricated flexible symmetric supercapacitor device that also provide reasonable energy density that may be helpful to enlighten the future flexible electronic world.

## 4 Experimental Section

The chemicals used in the experiment and the characterization techniques with specification of instruments are provided in the Supporting Information.

### Fabrication of Sheath‐Core Nanofibers

PAN (12 wt.%) and 26 wt.% PMMA solutions were prepared in DMF with stirring at 25 °C for 24 h. The PAN and PMMA solutions were supplied to the sheath‐core manufacturing nozzle in a 3:2 ratio for electrospinning. The distance between the nozzle and the tip was 20 cm, and electrospinning was performed at a high voltage of 15 kV and a humidity of 40% at room temperature. After electrospinning, the sheath‐core nanofiber membrane was dried in a vacuum oven to volatilize the solvent.

### Synthesis of ZIF‐67 on Sheath‐Core Nanofibers

The samples were prepared under three conditions. At first, 1, 1.5, and 2 g of cobalt nitrate were separately dissolved in distilled water. The prepared sheath‐core nanofiber membrane was then immersed in a cobalt nitrate solution. After that, an aqueous solution containing 2 g of 2‐methyl imidazole was added to the solution, in which the nanofibers were immersed and aged for 24 h. To improve the flexibility of the nanofibers, an aqueous solution containing 1 g of ammonium bicarbonate and 2 g of citric acid was used. The ammonium bicarbonate solution was poured into the cobalt nitrate solution, in which the sheath‐core nanofiber membrane was immersed, and then the citric acid solution was uniformly sprayed over the membrane. Finally, the nanofiber membrane was washed with distilled water and dried at 60 °C in vacuum.

### Growth of N‐CNTs on Hollow CNFs

ZIF‐67‐covered sheath‐core nanofibers were stabilized at 250 °C for 4 h under an oxygen atmosphere. After stabilization, carbonization was performed for 0.5 h at a carbonization temperature of 1000 °C under a nitrogen atmosphere. The N‐CNTs were grown uniformly, whereas PMMA was removed through the CNFs during the carbonization process to obtain CNT‐grown hollow CNFs. Finally, the samples were thoroughly washed with nitric acid to remove the surface Co‐NPs and to create defects and holes, which increased the capacitance of CNTs and enhanced their ion‐diffusion capacity, as proven theoretically by using density functional theory (DFT) calculations.^[^ ^63^ ^]^ The samples were named CNT@HCNF‐1, CNT@HCNF‐1.5, and CNT@HCNF‐2 according to the weight of cobalt nitrate used for ZIF‐67 preparation. The optimum carbonization temperature (1000 °C) was selected, as in our previous studies. Additionally, the homogeneous growth of CNTs was observed at this temperature, which also preserved the flexibility of nanofibrous membranes with a high porosity.

### Electrochemical Measurements

All electrochemical measurements were evaluated at room temperature in a VersaSTAT‐4 electrochemical workstation (AMETEK, Inc., USA). As an electrolyte, an aqueous KOH (2 m) solution was used for CV, GCD, and EIS tests in a three‐electrode system. Ag/AgCl was used as the reference electrode, and Pt wire was used as the counter electrode. EIS experiments were performed with a voltage amplitude of 5 mV at an open‐circuit potential from a frequency range of 10^5^–10^−2^ Hz. The GCD technique was used to calculate the specific capacitance of the electrode and fabricated SSC and ASC devices using Equation 1:

(3) $$C_{s}=\frac{I_{d}\times \Delta t}{\Delta V\times m}\;\;$$

where *C*_s_ is the specific capacitance in F g^–1^, *I*_d_ is the discharge current in amperes, Δ*t* represents the discharging time in seconds, Δ*V* is the working potential range in voltage, and *m* is the mass of the active materials in grams.

### Fabrication of the ASC and SSC Devices

A PVA/KOH gel was used as the electrolyte to fabricate both the SSC and ASC devices. In the case of an ASC device, a mass balance between positive and negative electrodes can be achieved according to Equation 2:

(4) $$Q_{+/-}=\;C_{s}\times \;m\;\times \;\Delta V$$

When Q_+_ = Q_–_, Equation 2 takes the form of Equation 3:

(5) $$\frac{m_{+}}{m_{-}}=\frac{C_{s-}\times \;\Delta V_{-}}{C_{s+}\times \;\Delta V_{+}}\;\;$$

Furthermore, the energy density (*E*) in W h kg^–1^ and the power density (*P*) in W kg^–1^ of the SSC and ASC devices were evaluated using Equations 4 and 5, respectively:

(6) $$E\;=\;\frac{1}{2\times \;3.6}C_{s}{\left(\Delta V\right)}^{2}\;$$

(7) $$P\;=\;\frac{E_{s}\times \;3600}{\Delta t}\;$$

where Δ*t* represents the discharging time in seconds.

### Statistics

The output performance of CV, GCD, and EIS (current, voltage, time, frequency, resistance) were all adopted directly from the measured original data using the abovementioned measurement machines. Based on the measured data, the current density, Energy density, power density, coulombic efficiency, and retention percentages were computed. All of the normalized data were normalized based on the highest value of each data. All of the curves were designed using Origin (software).

## Conflict of Interest

The authors declare no conflict of interest.

## Supporting information

Supporting Information

## Data Availability

The data that support the findings of this study are available on request from the corresponding author. The data are not publicly available due to privacy or ethical restrictions.
