# Supplementary material for: Homogeneous Elongation of N‐Doped CNTs over Nano‐Fibrillated Hollow‐Carbon‐Nanofiber: Mass and Charge Balance in Asymmetric Supercapacitors Is No Longer Problematic
Source: Adv Sci (Weinh). 2022 May 14;9(20):2200650. doi: 10.1002/advs.202200650 (PMC9284134; doi:10.1002/advs.202200650)
Supplement: Supplementary file 1 — Supporting Information [file ADVS-9-2200650-s001.pdf]

Supporting Information

Homogeneous elongation of N-doped CNTs over nano-fibrillated hollow-carbon-nanofiber: mass and charge balance in asymmetric supercapacitors is no longer problematic

Taewoo Kim, Subhangi Subedi, Bipeen Dahal, Kisan Chhetri, Tanka Mukhiya, Alagan Muthurasu, Jagadis Gautam, Prakash Chandra Lohani, Devendra Acharya, Ishwor Pathak, Su-Hyeong Chae, Tae Hoon Ko, Hak-Yong Kim**

T. Kim, S. Subedi, B. Dahal, K. Chhetri, T. Mukhiya, A. Muthurasu, P. C. Lohani, D. Acharya. I Pathak, S. H Chae, T. H. Ko, Prof. H. Y. Kim

Department of Nano Convergence Engineering

Jeonbuk National University, Jeonju, 54896, Republic of Korea

Email: khy@jbnu.ac.kr/dahalbipeen@gmail.com

S. Subedi

Department of Chemistry

Trichandra Multiple Campus

Tribhuvan University, Kathmandu, Nepal

B. Dahal

Central Department of Chemistry

Tribhuvan University, Kathmandu, Nepal

Email: dahalbipeen@gmail.com

T. Mukhiya

Department of Chemistry

Bhaktapur Multiple Campus

Tribhuvan University, Kathmandu, Nepal

P.C. Lohani, I. Pathak

Department of Chemistry

Amrit Campus

Tribhuvan University, Kathmandu, Nepal

J. Gautam

School of Materials Science and Engineering,

Kumoh National Institute of Technology, 61 Daehak-ro, Gumi-si, Gyeongsangbuk-do 39177, Republic of Korea.
Prof. H. Y. Kim
Department of Organic Materials and Fiber Engineering
Jeonnbuk National University, Jeonju 561-756, Republic of Korea
Email: khy@jbnu.ac.kr

Chemicals

Poly(acrylonitrile) (PAN (M_w = 150,000), poly(methyl methacrylate) (PMMA, M_w = 120,000) and 2-methylimidazole ($C_4H_6N_2$, 99%) were purchased from Sigma–Aldrich. N-Methyl-2-pyrrolidinone (C_5H_9NO), 99.5%) was purchased from Showa Chemicals Inc., Japan. Cobalt nitrate hexahydrate (Co $(NO_3)_2{\cdot}6H_2O$, 97%), potassium hydroxide (KOH, ≥99.5%), ammonium bicarbonate (NH_4HCO_3, 95%), N,N-dimethylformamide (DMF, $HCON(CH_3)_2$, 99.5%), and sulfuric acid (H_2SO_4, 95%) were purchased from Samchun, Republic of Korea. Methanol (CH_3OH, 99.9%) was purchased from Fisher Scientific, Republic of Korea. Commercially available materials and chemicals were used as received.

Characterizations

The X-ray diffraction (XRD) patterns were recorded using a Rigaku Ultima Plus X-ray diffractometer (Rigaku, Co., Japan) Morphology of the prepared samples were investigated by a field emission scanning electron microscope (Hitachi S-4800, Japan) equiped with energy dispersive X-ray spectrometer (EDX). Transmission electron microscopy (TEM) was performed on JEOL, JEM-2200FS (Japan) equipped with EDX. The same equipment was used to study high-resolution TEM (HR-TEM) and selected area electron diffraction (SAED). Chemical composition and bonding among the elements were studied using X-ray photoelectron spectrometry (XPS) in a X-ray photoelectron spectrometer (Thermo Fisher Scientific, USA). RFS-100S FT-Raman spectrometer (Germany) was used to acquire Raman spectra. N_2 adsorption experiments were performed using BELSORP-max adsorption analyser (Japan) to study Brunauer-Emmett-Teller (BET) parameters. Contact angle measurements were evaluated

using a standard Goniometer (200-F1, Rame-Hart Instrument Co., USA).

Mass balancing during ASC device fabrication

The mass ratio of active electrode materials in ASC device was achieved by using following mass balance equation;

$$\frac{m_+}{m_-} = \frac{C_- \times V_-}{C_+ \times V_+}$$

where m_+, m_-, C_+, C_-, V_+, and V_- are the mass of active materials (g), specific capacitance (F g^{-1}), and potential window (V) of positive and negative electrodes, respectively. Mass balance was achieved using CNT@HCNF-1.5 as active material in the negative electrode.

Based on the calculations, the ratio of m_-:m_+ was fixed at 1:1.

Mass Balance Calculation

$C_+ = 1790$ F g^{-1}

$C_- = 712$ F g^{-1}

$V_+ = 0.4$ V

$V_- = 1$ V

Therefore, $m_+/m_- = 1:1.0056$ (~ 1:1)

Mass of CNT@HCNF-1.5 (1 cm^2) = 0.0025 g

Mass of active material (Co_3O_4) on positive electrode (m_+) = 0.0025 g

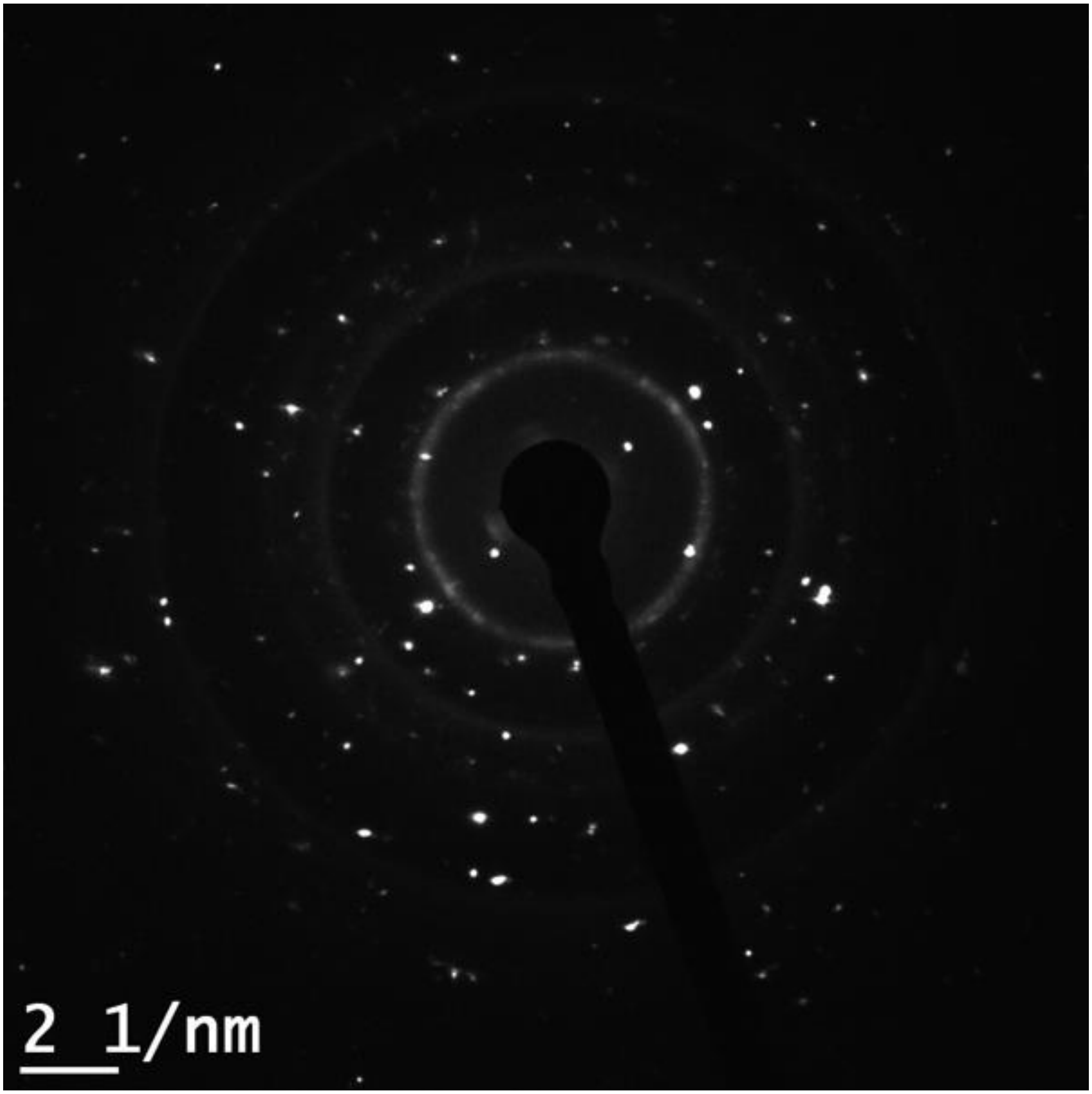

Figure S1. SAED pattern of CNT@HCNF-1.5.

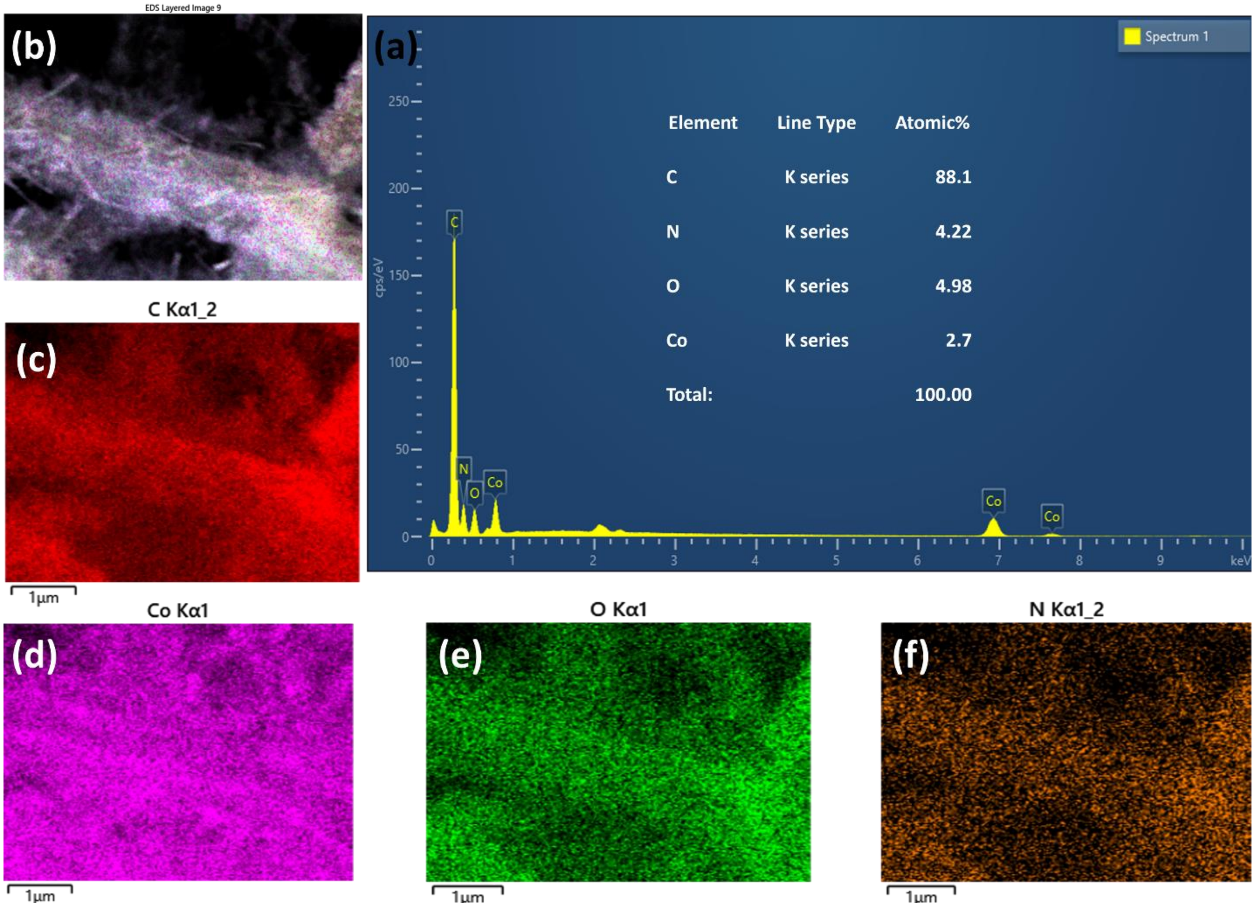

Figure S2. EDX analysis of CNT@HCNF-1.5, (a) EDX spectrum (inset: atomic percentages of the different elements), (b) sum elemental mapping spectrum, and elemental mapping spectra of (c) C, (d) Co, (e) O, and (f) N.

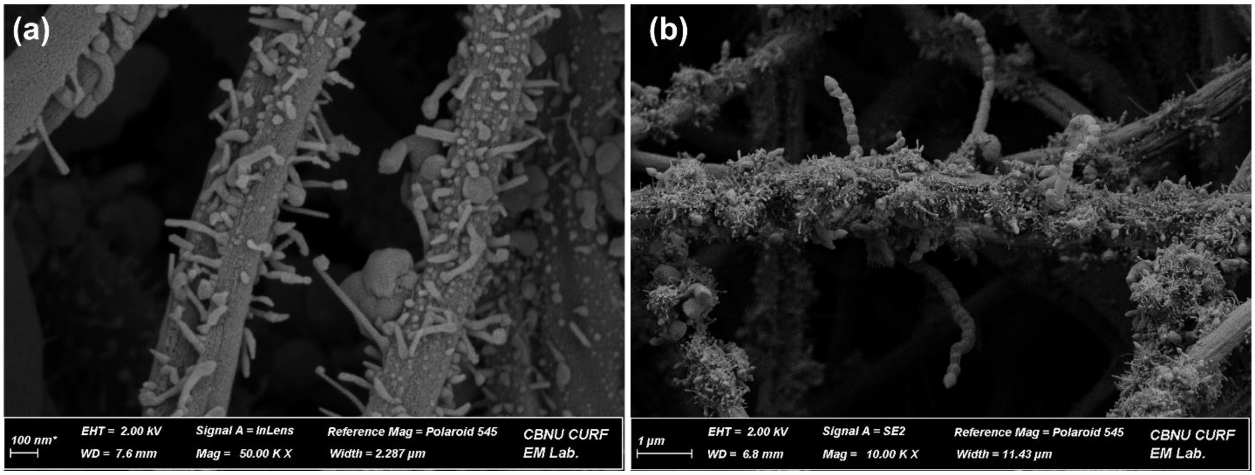

Figure S3. FE-SEM images of (a) CNT@HCNF-1, and (b) CNT@HCNF-2.

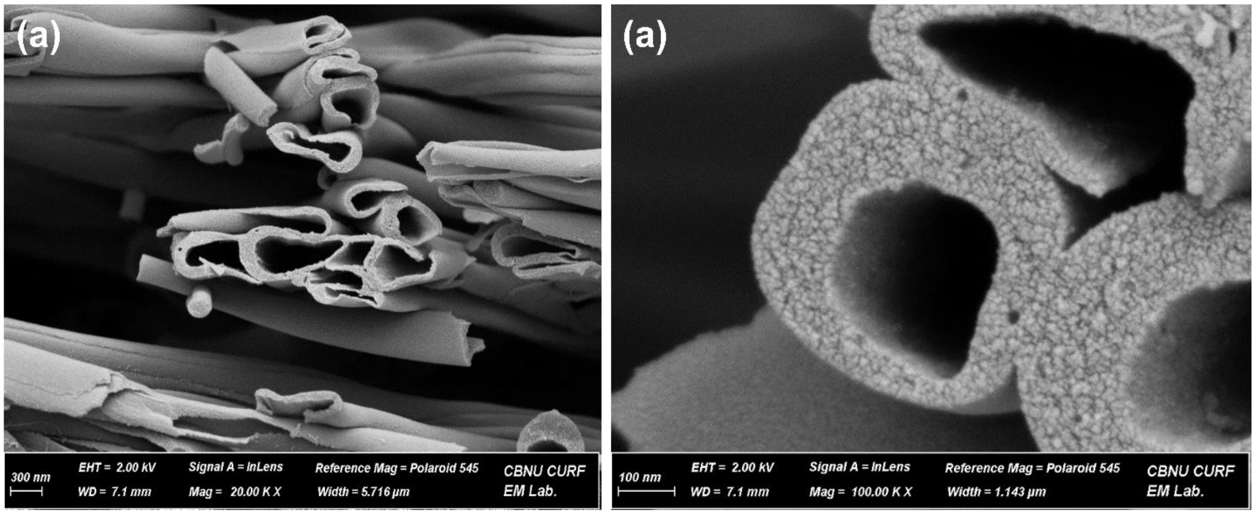

Figure S4. FE-SEM images of nano-fibrillated HCNF.

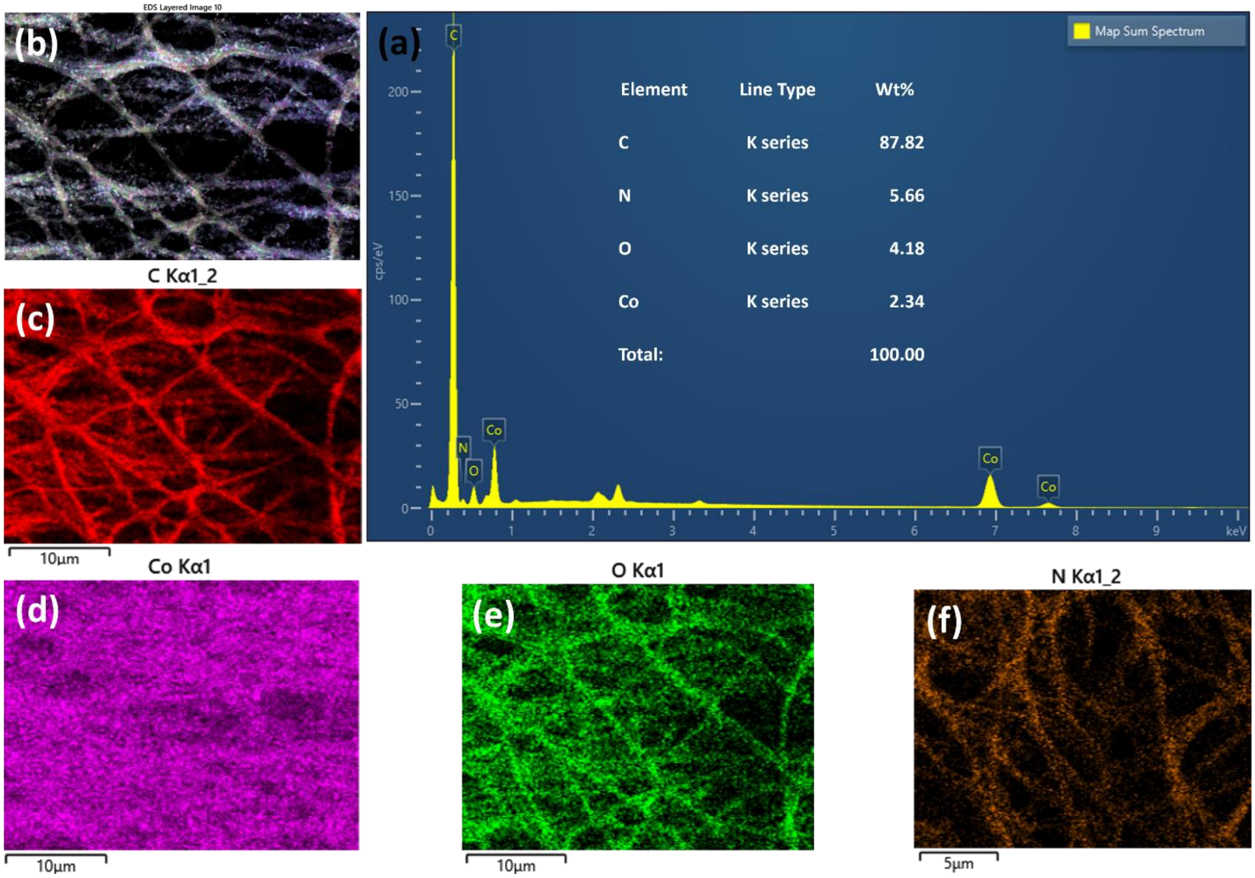

Figure S5. EDX analysis of CNT@HCNF-1, (a) EDX spectrum (inset: atomic percentages of the different elements), (b) sum elemental mapping spectrum, and elemental mapping spectra of (c) C, (d) Co, (e) O, and (f) N.

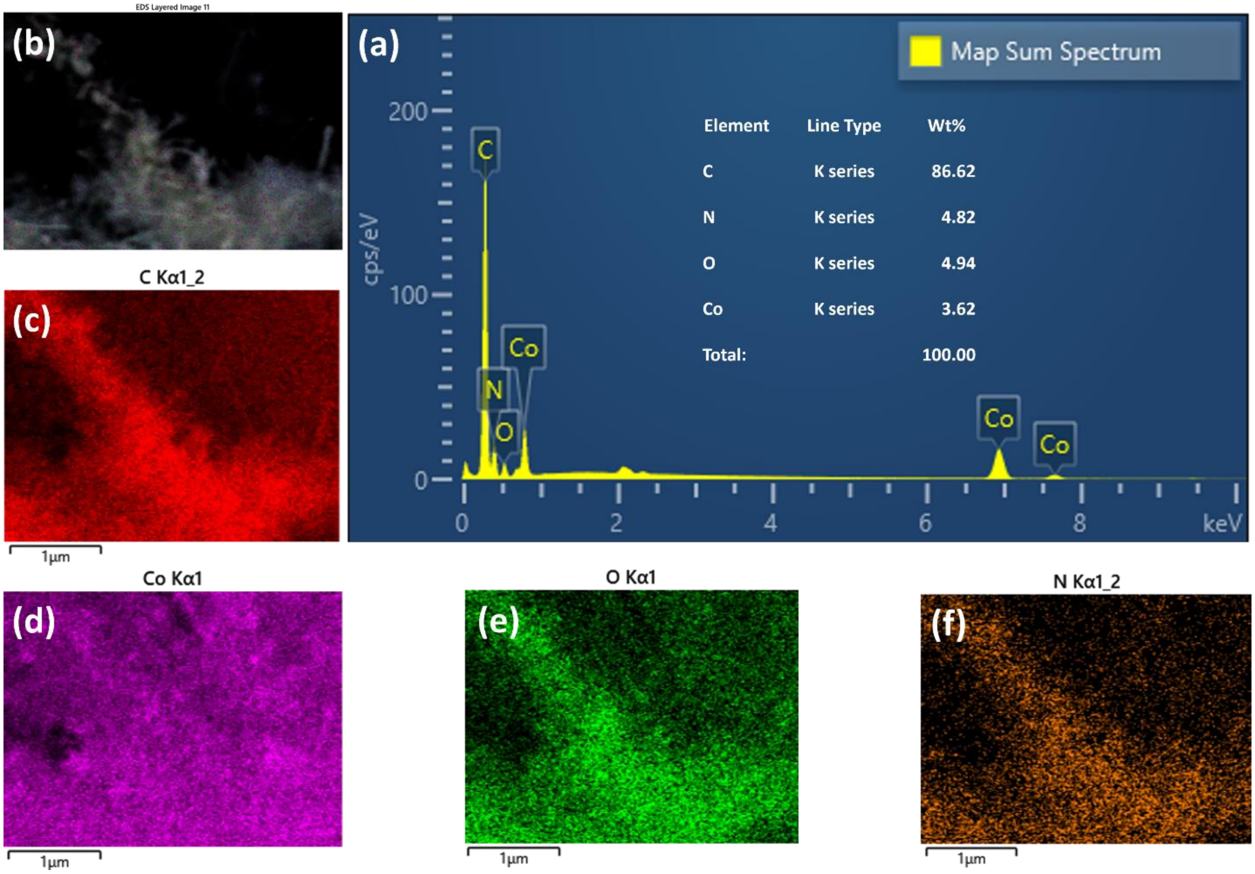

Figure S6. EDX analysis of CNT@HCNF-2: (a) EDX spectrum (inset: atomic percentages of the different elements), (b) sum elemental mapping spectrum, and elemental mapping spectra of (c) C, (d) Co, (e) O, and (f) N.

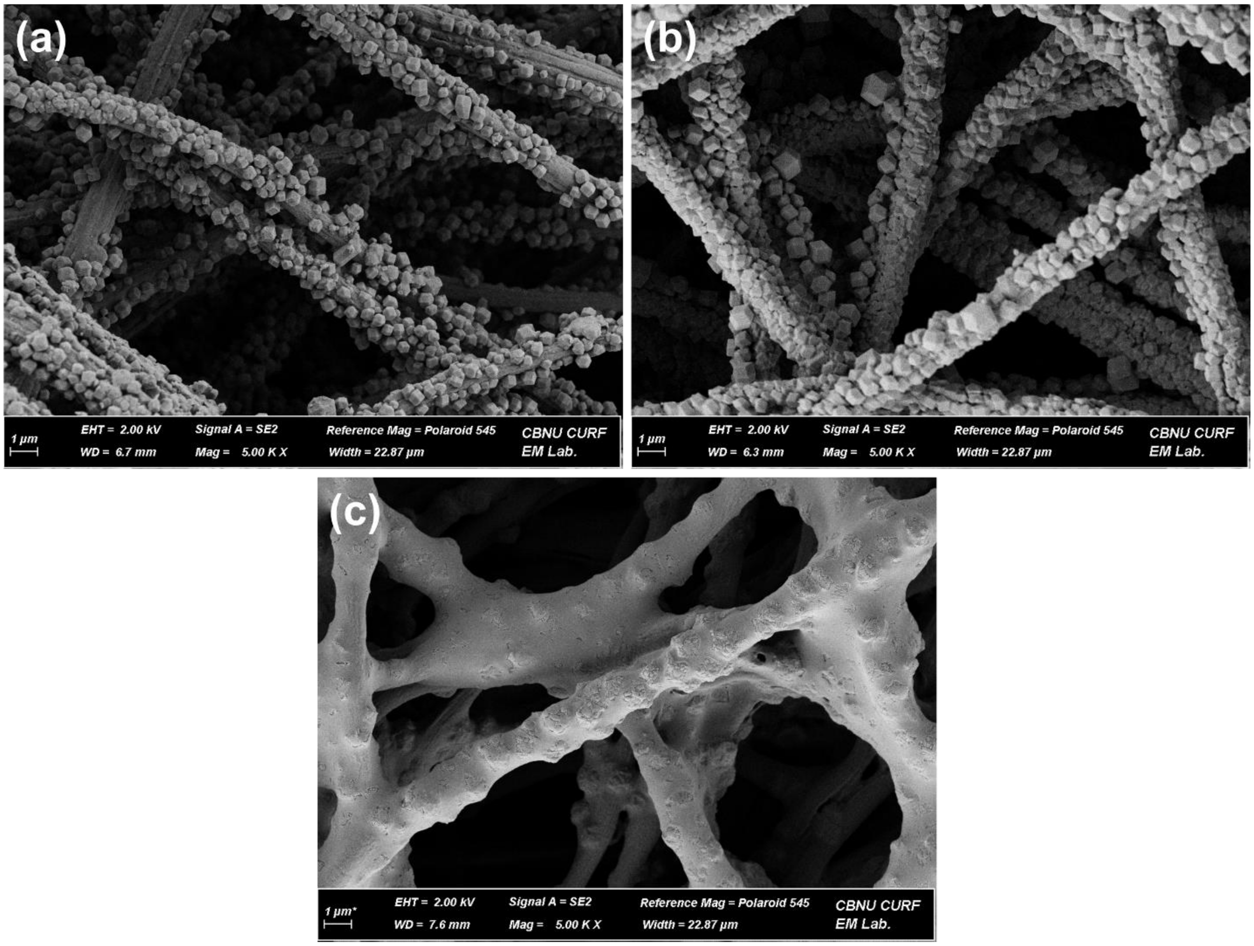

Figure S7. Representative FE-SEM images of (a) ZIF-67@PAN/PMMA-1 (b) ZIF-67@PAN/PMMA-1.5 (c) ZIF-67@PAN/PMMA-2.

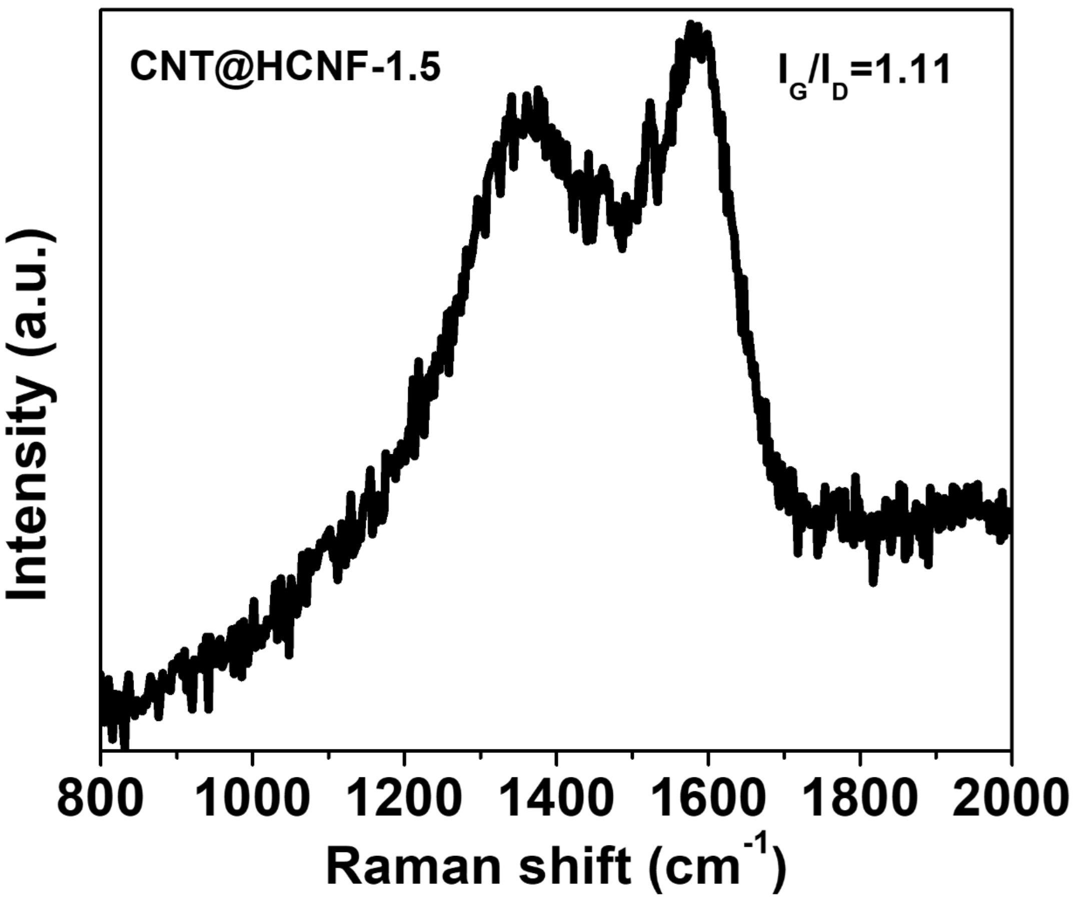

Figure S8. Raman Spectrum of CNT@HCNF-1.5.

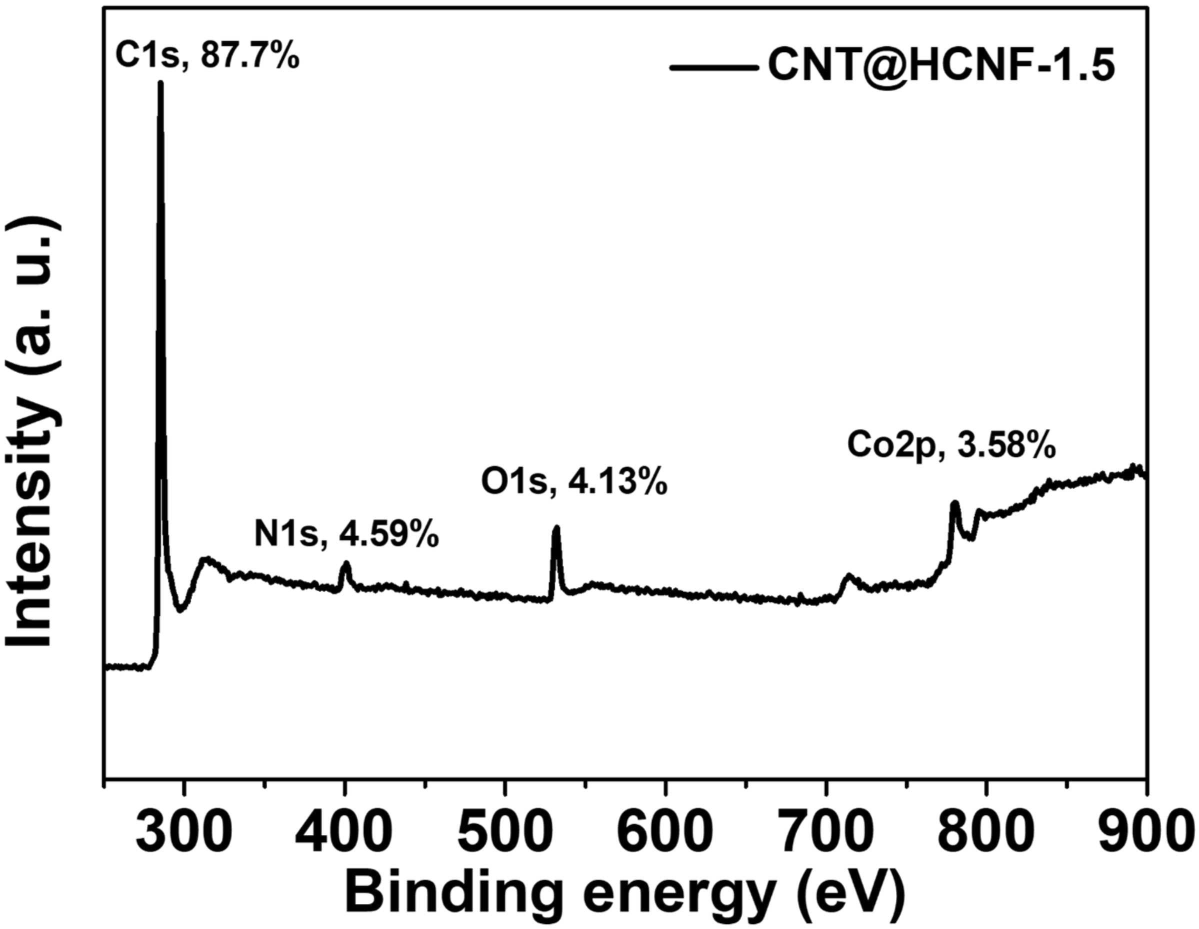

Figure S9. Low resolution XPS spectrum with percentage of the respective elements.

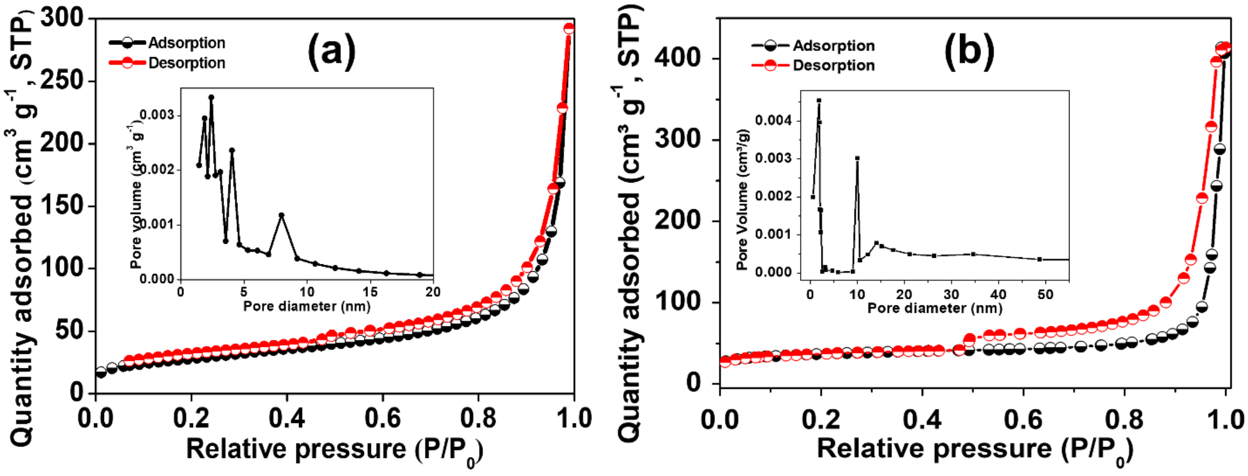

Figure S10. Adsorption isotherms and pore size distribution (inset) of (a) CNT@HCNF-1, and (b) CNT@HCNF-2.

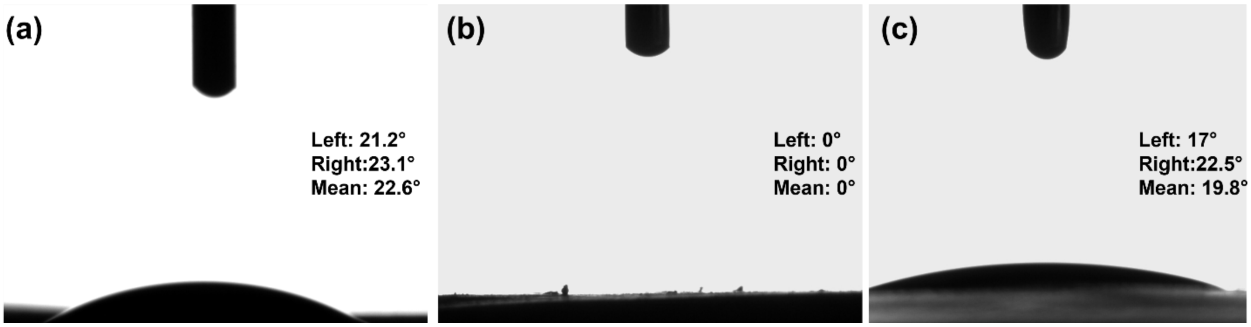

Figure S11. Contact angle measurements of (a) CNT@HCNF-1, (b) CNT@HCNF-1.5, and (c) CNT@HCNF-2 in 2 M KOH

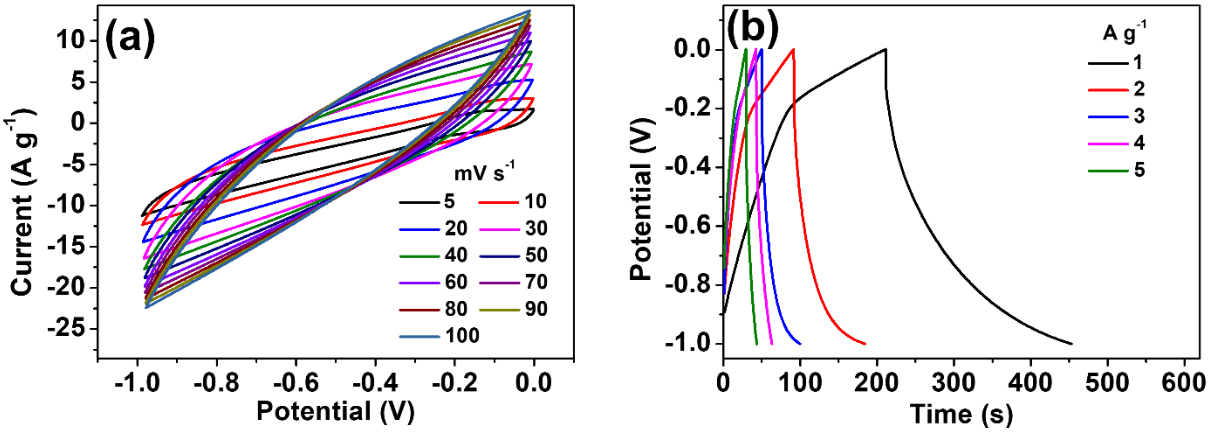

Figure S12. Electrochemical performances of CNT@HCNF-1. (a) CV curves, and (b) GCD curves.

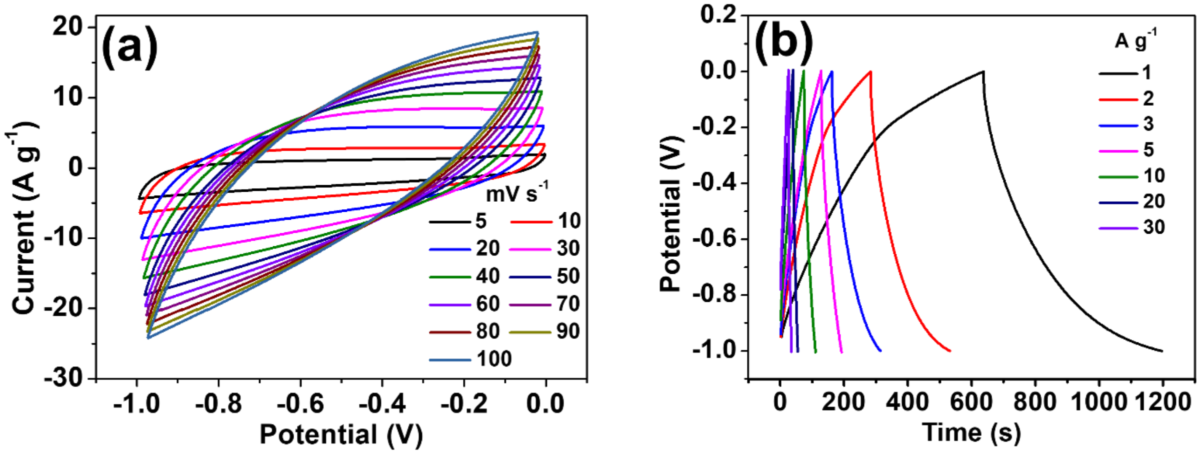

Figure S13. Electrochemical performances of CNT@HCNF-2. (a) CV curves, and (b) GCD curves.

The storage mechanism of an EDLC type electrode material is electrostatic adsorption, known as capacitive non-Faradaic charge storage and the capacitance is determined by

$$C = \frac{\Delta Q}{\Delta V} = i\ \frac{\Delta t}{\Delta V} \qquad (5)$$

However, for the materials based on Faradic charge storage behavior, the above definition is not appropriate to describe the system rather the term capacity is mostly used in place of capacitance. In case of CNT@HCNF-1.5 electrode the charge storage mechanism is dominated by capacitive non-Faradic type, the term capacitance has been used to evaluate the capacitive performance throughout the manuscript.

The storage mechanism of CNT@HCNF-1.5 major electrode has been studied using the relation $i = av^b$. To confirm the charge storage kinetics of electrode, the logarithm relationship between the peak current density (i) and scan rate (v) is used to distinguish whether the electrochemical process is diffusion-controlled or surface-controlled.

$$i=av^b \qquad (1)$$

$$\log(i)=b\log(v)+\log(a) \qquad (2)$$

where, a and b are the constants. The value of b is equal to 0.5 indicates diffusion-controlled electrochemical process due to the Faradaic intercalation reaction and a b-value of 1.0 demonstrates a totally capacitive non-Faradic behavior due to surface capacitive effect. For CNT@HCNF-1.5, the calculated b value is closer to 1 (b = 0.952) indicating the dominance of capacitive non-Faradic type storage mechanism. The storage contribution ratio between two types of electrochemical processes at a particular scan rate and potential can be calculated from the following equations:

$$i\ (V)=k_1v+k_2v^{1/2} \qquad (3)$$

$$i\ (V)/v^{1/2}=k_1v^{1/2}+k_2 \qquad (4)$$

where, k_1v represents capacitive non-Faradic contribution and $k_2v^{1/2}$ represents diffusion controlled faradic contribution. From calculations, the capacitive non-Faradic contribution was ~80% at scan rate of 5 mV s^{-1} which increased to ~93% at 100 mV s^{-1}.

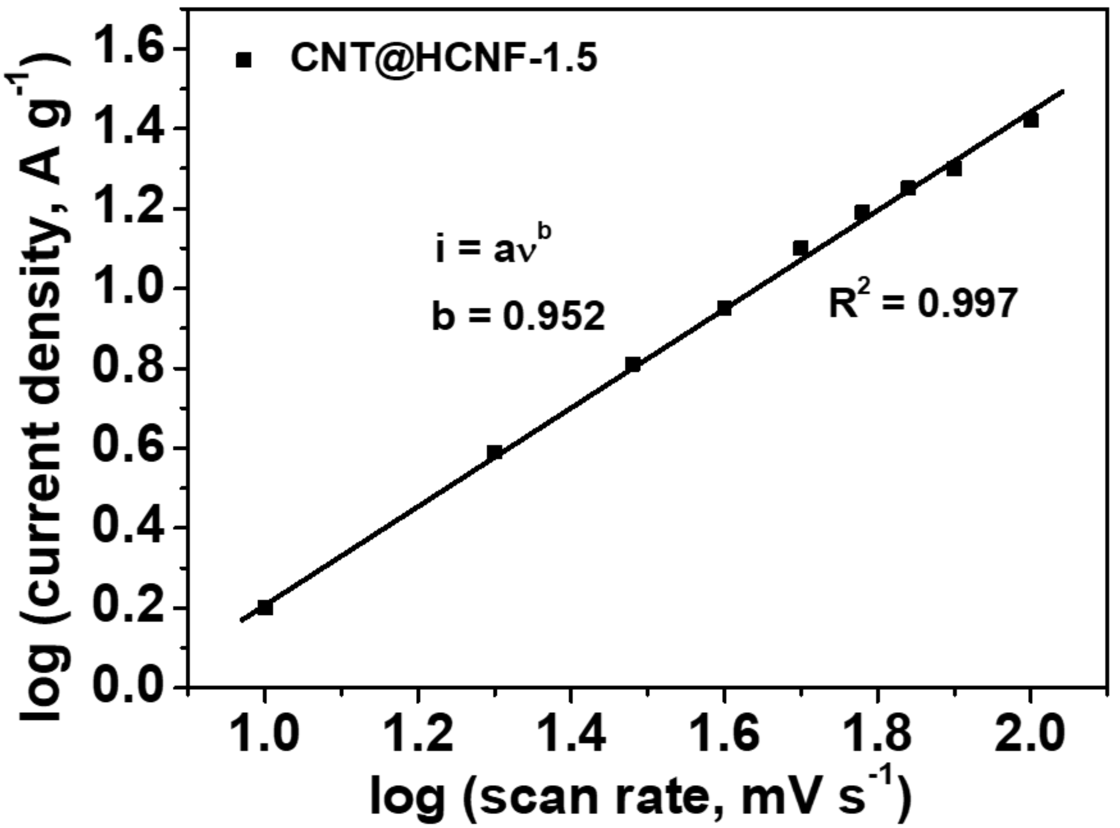

Figure S14. The plots of log(current density,i) against log(scan rate,v).

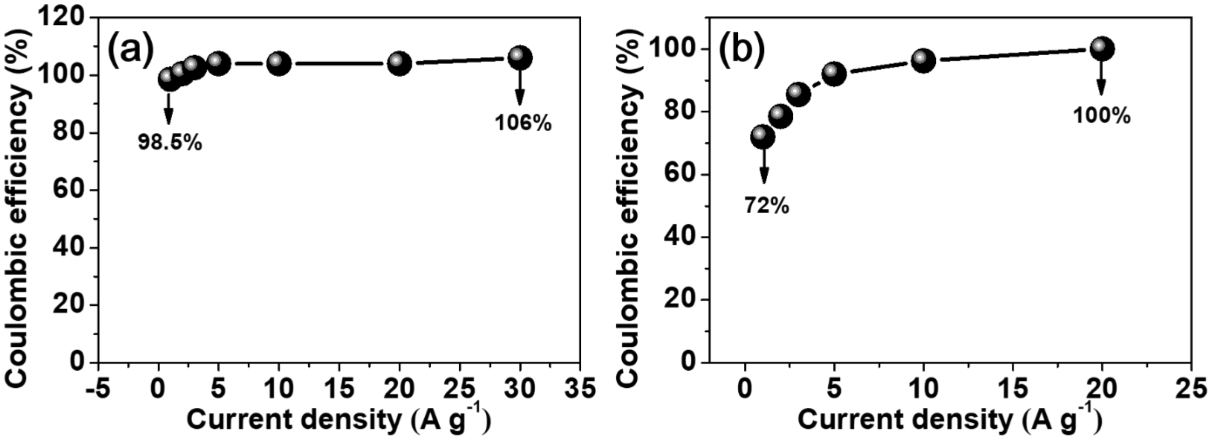

Figure S15. Coulombic efficiencies of (a) CNT@HCNF-1.5 negative electrode and (b) ASC device at different current densities.

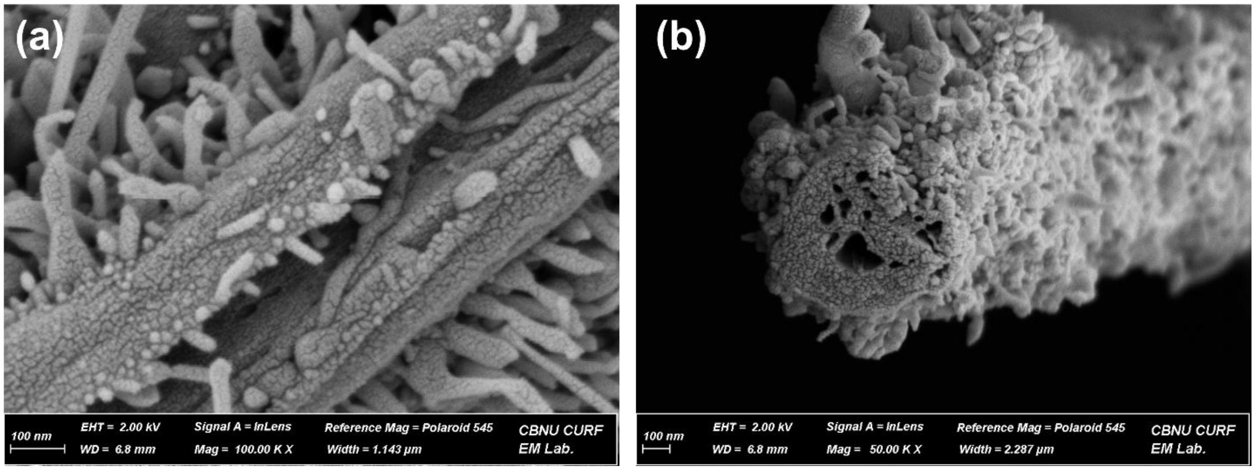

Figure S16. FE-SEM images of CNT@HCNF-1.5 after the stability test.

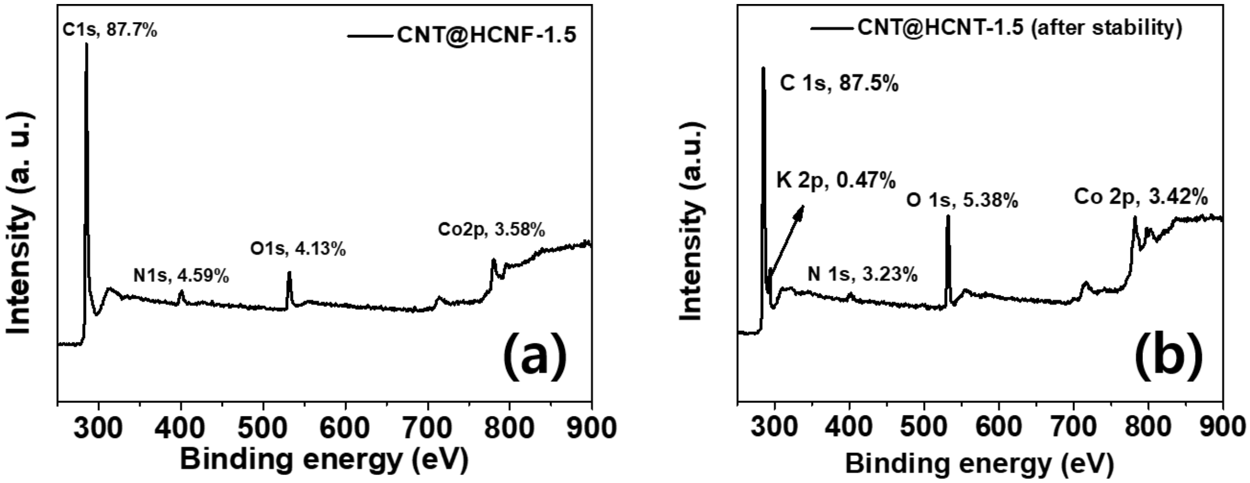

Figure S17. XPS survey spectrum of CNT@HCNF-1.5; (a) before and (b) after the stability test.

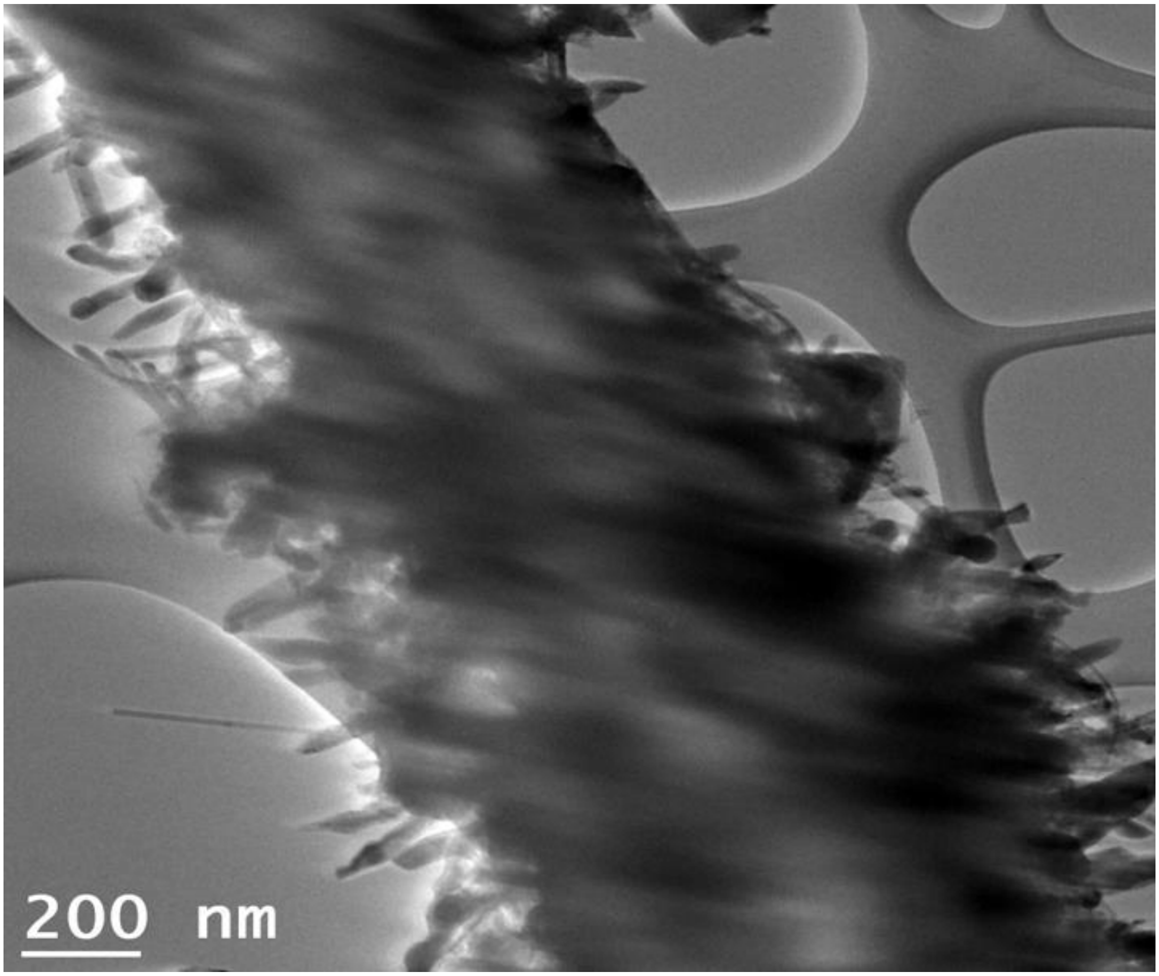

Figure S18. TEM image of CNT@HCNF-1.5 after the stability test.

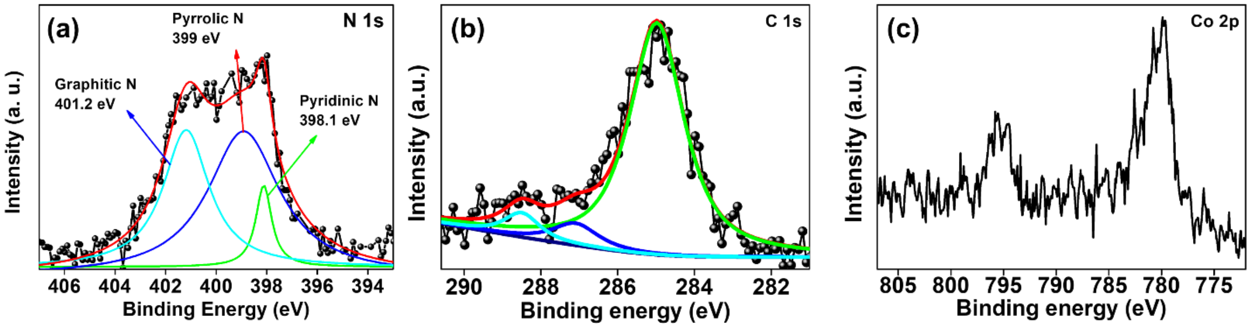

Figure S19. Deconvoluted high-resolution XPS spectra of CNT@HCNF-1.5 for (a) N, (b) C, and (c) Co after the stability test. In the case of nitrogen, the oxidized nitrogen peak could not be fitted after the stability test.

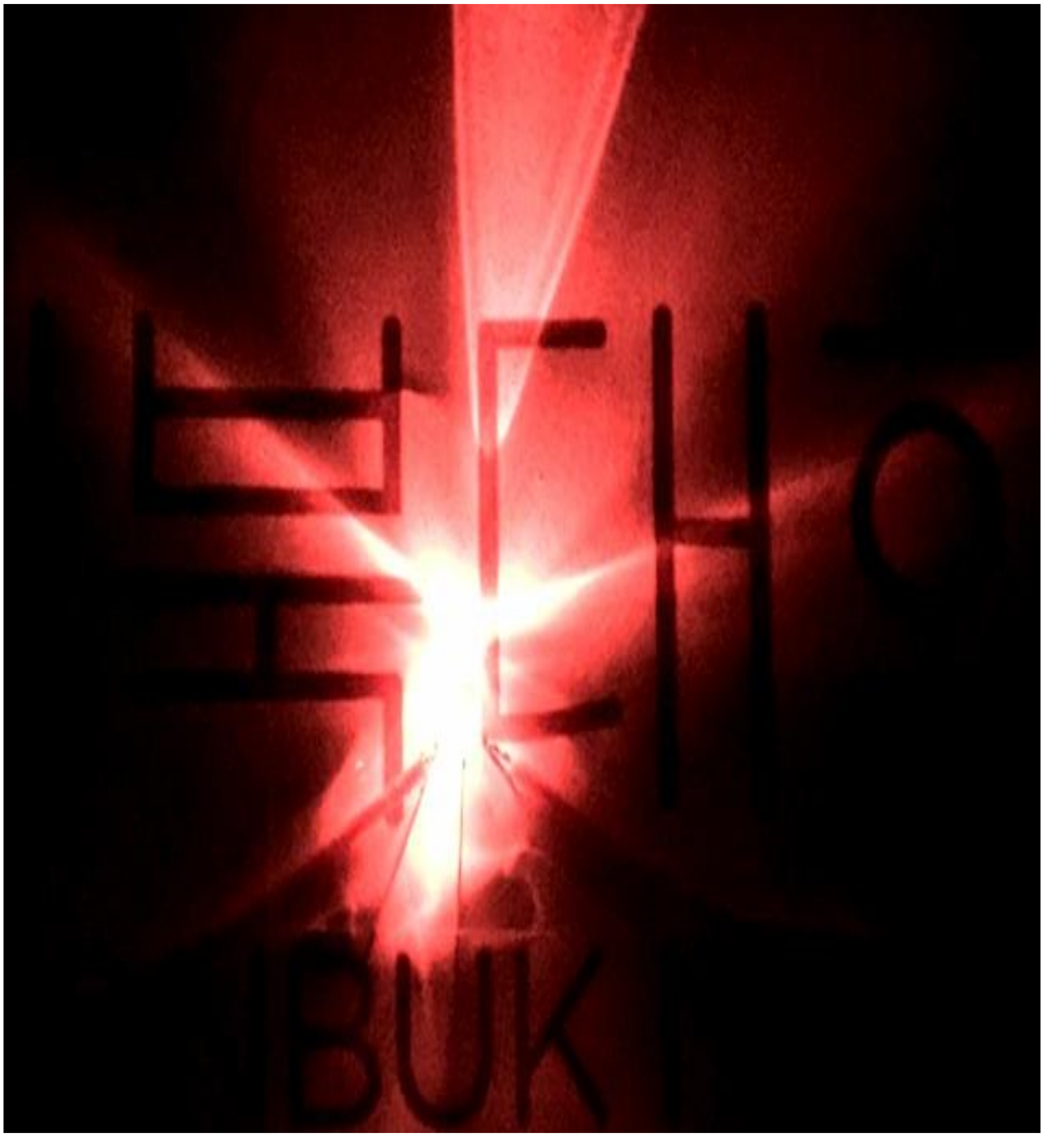

Figure S20. A 1 W red LED powered by two flexible SSC devices connected in series (after charging) for approximately 20 min at 20 A g^{-1}, demonstrating the practical applicability of the device.

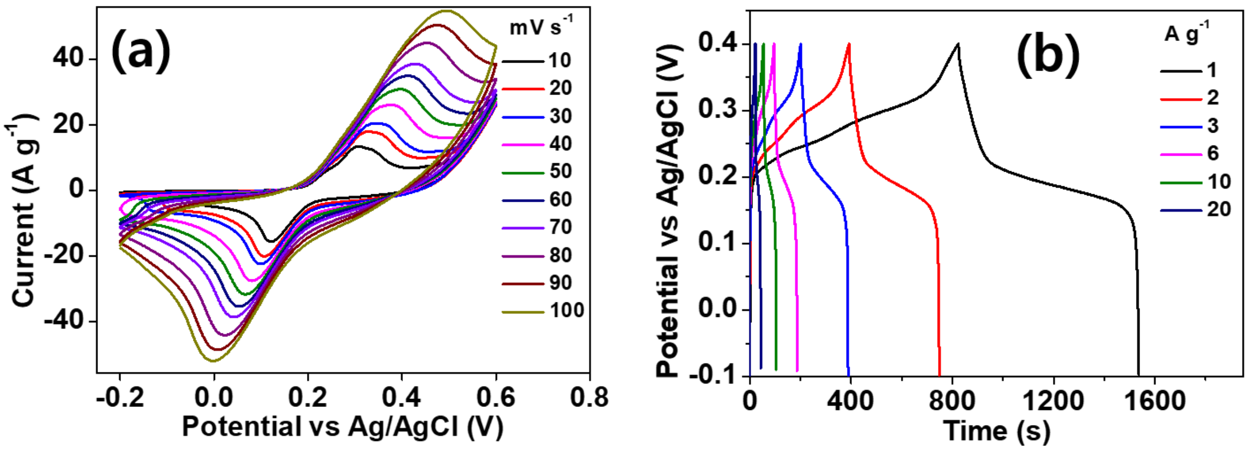

Figure S21. Electrochemical performance of the Co_3O_4@NF positive electrode. (a) CV curves, and (b) GCD curves. At 1 A g^{-1}, Co_3O_4@NF furnished a capacitance of 1790 F g^{-1}.

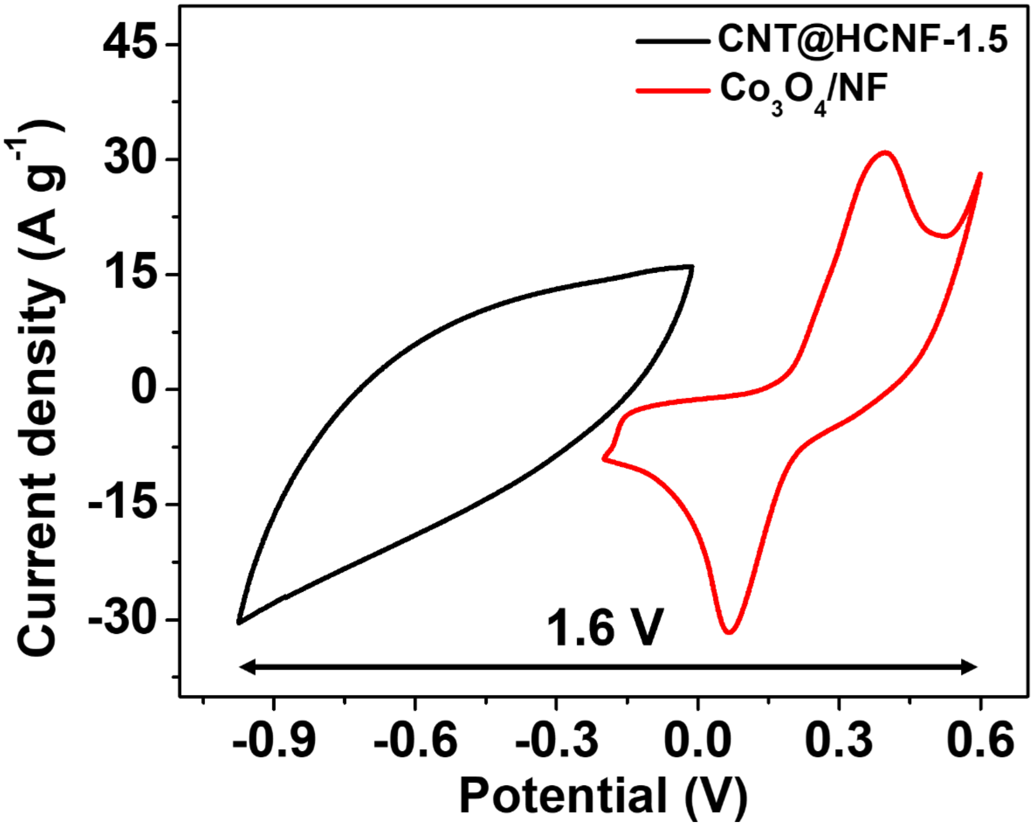

Figure S22. CV curves of the negative and positive electrodes showing the extension of the working potential of the ASC device up to 1.6 V.

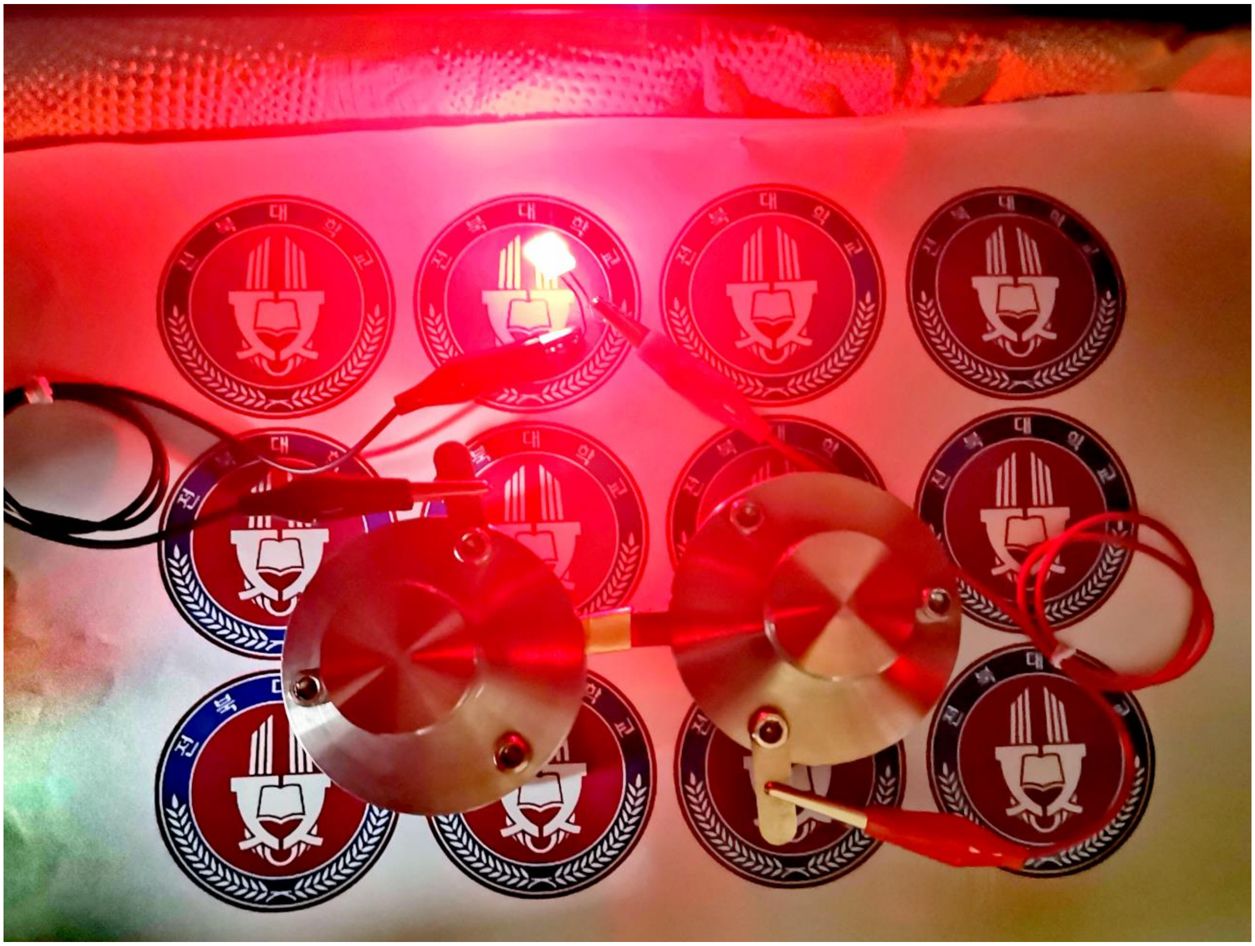

Figure S23. A red LED powered by two ASC devices connected in series (after charging) for approximately 12 min at 10 A g^{-1}, demonstrating the practical applicability of the device.

Table S1. BET Texture properties of different samples.

**Samples**	**BET surface area, S_{BET} (m^2 g^{-1})**	**Total pore volume, V_T (cm^3 g^{-1})**	**Average pore diameter, R_{av} (nm)**
HCNF	79.2	0.23	4.1
CNT@HCNF-1	386.2	0.66	4.5
CNT@HCNF-1.5	812.6	1.18	9.8
CNT@HCNF-2	668.2	0.92	9.4

Table S2. Electrical conductivities of CNT@HCNF-1, CNT@HCNF-1.5 and CNT@HCNF-2 along with HCNF fiber mats.

**Sample**	**Electrical conductivity (S m^{-1})**
**CNT@HCNF-1**	442 S m^{-1}
**CNT@HCNF-1.5**	496 S m^{-1}
**CNT@HCNF-2**	416 S m^{-1}
**HCNF**	105 S m^{-1}

Table S3. Nyquist impedance parameters of the different electrodes.

**Sample**	**R_s (Ω)**	**R_{ct} (Ω)**	**W (Ω)**
CNT@HCNF-1	0.44	0.09	0.08
CNT@HCNF-1.5	0.45	0.06	0.07
CNT@HCNF-2	0.48	0.1	0.08

Table S4. Nyquist impedance parameters of the fabricated SSC device.

**Device**<br>CNT@HCNF-1.5//CNT@HCNF-1.5	**R_s (Ω)**	**R_{ct} (Ω)**	**W (Ω)**	**ESR (Ω)**
before stability	0.16	0.08	0.05	0.29
after stability	0.21	0.11	0.05	0.37

Table S5. Nyquist impedance parameters of the fabricated ASC device.

**Device**<br>Co_3O_4@NF//CNT@HCNF-1.5	**R_s (Ω)**	**R_{ct} (Ω)**	**W (Ω)**	**ESR (Ω)**
Before stability	0.68	1.99	0.16	2.83
After stability	0.74	2.26	0.18	3.18

Table S6. Comparison of the electrochemical performances of CNT@HCNF-1.5 with similar electrode materials.

**Electrode materials**	**Capacitance, F g^{-1}**	**Current density, A g^{-1}**	**Electrolyte**	**Reference**
MOF-derived Hollow particles-based N-doped CNFs	307.2	1	2 M H_2SO_4	[1]
Graphene quantum dot reinforced Electrospun CNFs	355	1	6 M KOH	[2]
Tubular architectures with hollow N-doped carbon polyhedrons	351.2	1	1 M H_2SO_4	[3]
Porous N-doped carbon nanorods derived from ZIF-8	292.2	0.5	1 M H_2SO_4	[4]
Porous N-doped carbon/CNT	250	1	1 M Na_2SO_4	[5]
B, N-co-doped 3D porous CNFs	295	0.5	2 M KOH	[6]
Polyphosphazene derived N, P, O-doped porous carbon	339	0.5	6 M KOH	[7]
Biomass derived 3D hierarchical porous carbon nanosheets	588	0.5	6 M KOH	[8]
Graphene/CNFs/CNTs	521.5	0.25	6 M KOH	[9]
CNTs on CNFs	464.2	0.5	6 M KOH	[10]
CNTs on graphene	401	1	6 M KOH	[11]
Hierarchical porous N-doped CNTs	365.9	0.1	6 M KOH	[12]
Porous flexible CNFs	362	0.2	6 M KOH	[13]
ZIF-8 nanoporous carbon	267	1	3 M KOH	[14]
Microporous CNFs	316	0.2	6 M KOH	[15]
N-self-doped porous carbon	332	0.5	6 M KOH	[16]

N/B co-doped ordered mesoporous carbon spheres	272	0.5	6 M KOH	[17]
P-doped thick carbon electrode	384	1	6 M KOH	[18]
**CNT@HCNF-1.5**	**712**	**1**	**2 M KOH**	**This work**

Table S7. Comparison of the energy and power densities of the CNT@HCNF-1.5//CNT@HCNF-1.5 symmetric device with similar symmetric devices.

**Device (symmetric)**	**Energy density, Wh kg^{-1}**	**Power density, W kg^{-1}**	**Electrolyte**	**Reference**
N, P, O doped carbon	3.6	74	6 M KOH	[7]
N, S Co-doped porous carbon fiber	16.3	147.15	PVA/KOH gel	[19]
Ultrathin carbon gauze	4.22	380	6 M KOH	[20]
Lignocellulose-derived porous phosphorus-doped carbon	4.7	833	6 M KOH	[21]
O, N, S enriched activated carbon	21.48	14000	1 M $NaClO_4$	[22]
Porous hollow CNFs	12.99	600	6 M KOH	[23]
Porous multichannel CNFs	18.8	400	1 M H_2SO_4	[24]
N, P doped CNFs derived from bacterial cellulose	7.76	150	2 M H_2SO_4	[25]
Flexible and cross-linked N-doped CNFs	5.9	1200	1 M H_2SO_4	[26]
**CNT@HCNF-1.5//CNT@HCNF-1.5**	**20.13**	**5000**	**PVA/KOH gel**	**This work**

Table S8. Comparison of the energy and power densities of Co_3O_4/NF//CNT@HCNF-1.5 asymmetric devices with similar asymmetric devices.

**CO_3O_4//carbon-based ASC devices**	**Energy density, Wh kg^{-1}**	**Power density, W kg^{-1}**	**Electrolyte**	**Reference**
3D Co_3O_4/C@HCNFs//NGH	36.6	471	2 M KOH	[27]
Co_3O_4/NHCS//AC	34.5	735	2 M KOH	[28]
n-Co_3O_4/CC//NC	45.3	915	PVA/LiOH	[29]
Co_3O_4//AC	46.5	790.7	2 M KOH	[30]
Graphene/Co_3O_4//graphene	67.5	800	6 M KOH	[31]
3D Co_3O_4–RGO aerogel//HPC	40.65	340	6 M KOH	[32]
Co_3O_4//AC	24.9	225	6 M KOH	[33]
Vanadium-doped Co_3O_4//AC	66.88	240	3 M KOH	[34]
N-Co_3O_4@CF//Fe_3O_4@CF	39.7	222	PVA/KOH	[35]
**Co_3O_4/NF//CNT@HCNF-1.5**	**87.5**	**16000**	**PVA/KOH**	**This work**

References

[1] Zuo, W.; Li, R.; Zhou, C.; Li, Y.; Xia, J.; Liu, J., *Advanced science* **2017,** *4* (7), 1600539.

[2] Zhao, J.; Zhu, J.; Li, Y.; Wang, L.; Dong, Y.; Jiang, Z.; Fan, C.; Cao, Y.; Sheng, R.; Liu, A.; Zhang, S.; Song, H.; Jia, D.; Fan, Z., *ACS Applied Materials & Interfaces* **2020,** *12* (10), 11669-11678. DOI 10.1021/acsami.9b22408.

[3] Xu, H.; Yao, Z.; Gong, Y.; Dai, H.; Yu, C.; Qin, G.; Zhou, J.; Chen, Q.; Liu, X.; Sun, G., *Advanced Materials Interfaces* **2021,** *8* (16), 2100805. DOI https://doi.org/10.1002/admi.202100805.

[4] Keum, C.; Lee, H.; Kwon, C.; Han, B.; Lee, S.-Y., *Chemistry of Materials* **2020,** *32* (18), 7941-7950. DOI 10.1021/acs.chemmater.0c02782.

[5] Zhang, Y.; Lin, B.; Wang, J.; Tian, J.; Sun, Y.; Zhang, X.; Yang, H., *Journal of Materials Chemistry A* **2016,** *4* (26), 10282-10293. DOI 10.1039/C6TA03633C.

[6] Dahal, B.; Mukhiya, T.; Ojha, G. P.; Muthurasu, A.; Chae, S.-H.; Kim, T.; Kang, D.; Kim, H. Y., *Electrochimica Acta* **2019,** *301*, 209-219. DOI https://doi.org/10.1016/j.electacta.2019.01.171.

[7] Liu, W.; Zhang, S.; Dar, S. U.; Zhao, Y.; Akram, R.; Zhang, X.; Jin, S.; Wu, Z.; Wu, D., *Carbon* **2018,** *129*, 420-427. DOI https://doi.org/10.1016/j.carbon.2017.12.016.

[8] Selvaraj, A. R.; Muthusamy, A.; Inho, C.; Kim, H.-J.; Senthil, K.; Prabakar, K., *Carbon* **2021,** *174*, 463-474. DOI https://doi.org/10.1016/j.carbon.2020.12.052.

[9] Kshetri, T.; Tran, D. T.; Nguyen, D. C.; Kim, N. H.; Lau, K.-t.; Lee, J. H., *Chemical Engineering Journal* **2020,** *380*, 122543. DOI https://doi.org/10.1016/j.cej.2019.122543.

[10] Kshetri, T.; Thanh, T. D.; Singh, S. B.; Kim, N. H.; Lee, J. H., *Chemical Engineering Journal* **2018,** *345*, 39-47. DOI https://doi.org/10.1016/j.cej.2018.03.143.

[11] Yang, Z.-Y.; Zhao, Y.-F.; Xiao, Q.-Q.; Zhang, Y.-X.; Jing, L.; Yan, Y.-M.; Sun, K.-N., *ACS Applied Materials & Interfaces* **2014,** *6* (11), 8497-8504. DOI 10.1021/am501362g.

[12] Zhu, T.; Zhou, J.; Li, Z.; Li, S.; Si, W.; Zhuo, S., *Journal of Materials Chemistry A* **2014,** *2* (31), 12545-12551. DOI 10.1039/C4TA01465K.

[13] Ma, C.; Li, Y.; Shi, J.; Song, Y.; Liu, L., *Chemical Engineering Journal* **2014,** *249*, 216-225. DOI https://doi.org/10.1016/j.cej.2014.03.083.

[14] Chhetri, K.; Dahal, B.; Mukhiya, T.; Tiwari, A. P.; Muthurasu, A.; Kim, T.; Kim, H.; Kim, H. Y., *Carbon* **2021,** *179*, 89-99. DOI https://doi.org/10.1016/j.carbon.2021.04.028.

[15] Sheng, J.; Ma, C.; Ma, Y.; Zhang, H.; Wang, R.; Xie, Z.; Shi, J., *International Journal of Hydrogen Energy* **2016,** *41* (22), 9383-9393. DOI https://doi.org/10.1016/j.ijhydene.2016.04.076.

[16] Gong, Y.; Li, D.; Fu, Q.; Zhang, Y.; Pan, C., *ACS Applied Energy Materials* **2020,** *3* (2), 1585-1592. DOI 10.1021/acsaem.9b02077.

[17] Du, J.; Zhang, Y.; Lv, H.; Chen, A., *Journal of Colloid and Interface Science* **2021,** *587*, 780-788. DOI https://doi.org/10.1016/j.jcis.2020.11.037.

[18] Wang, F.; Cheong, J. Y.; He, Q.; Duan, G.; He, S.; Zhang, L.; Zhao, Y.; Kim, I.-D.; Jiang, S., *Chemical Engineering Journal* **2021,** *414*, 128767. DOI https://doi.org/10.1016/j.cej.2021.128767.

[19] Chen, L.; Wen, Z.; Chen, L.; Wang, W.; Ai, Q.; Hou, G.; Li, Y.; Lou, J.; Ci, L., *Carbon* **2020,** *158*, 456-464. DOI https://doi.org/10.1016/j.carbon.2019.11.012.

[20] Li, Z.; Li, L.; Li, Z.; Liao, H.; Zhang, H., *Electrochimica Acta* **2016,** *222*, 990-998. DOI https://doi.org/10.1016/j.electacta.2016.11.067.

[21] Yi, J.; Qing, Y.; Wu, C.; Zeng, Y.; Wu, Y.; Lu, X.; Tong, Y., *Journal of Power Sources* **2017,** *351*, 130-137. DOI https://doi.org/10.1016/j.jpowsour.2017.03.036.

[22] Manikandan, R.; Raj, C. J.; Moulton, S. E.; Todorov, T. S.; Yu, K. H.; Kim, B. C., *Chemistry – A European Journal* **2021,** *27* (2), 669-682. DOI https://doi.org/10.1002/chem.202003253.

[23] Liu, Y.; Liu, Q.; Wang, L.; Yang, X.; Yang, W.; Zheng, J.; Hou, H., *ACS Applied Materials & Interfaces* **2020,** *12* (4), 4777-4786. DOI 10.1021/acsami.9b19977.

[24] Ishita, I.; Singhal, R., *Journal of Applied Electrochemistry* **2020,** *50* (8), 809-820. DOI 10.1007/s10800-020-01433-0.

[25] Chen, L.-F.; Huang, Z.-H.; Liang, H.-W.; Gao, H.-L.; Yu, S.-H., *Advanced Functional Materials* **2014,** *24* (32), 5104-5111. DOI https://doi.org/10.1002/adfm.201400590.

[26] Cheng, Y.; Huang, L.; Xiao, X.; Yao, B.; Yuan, L.; Li, T.; Hu, Z.; Wang, B.; Wan, J.; Zhou, J., *Nano Energy* **2015,** *15*, 66-74. DOI https://doi.org/10.1016/j.nanoen.2015.04.007.

[27] Mukhiya, T.; Ojha, G. P.; Dahal, B.; Kim, T.; Chhetri, K.; Lee, M.; Chae, S.-H.; Muthurasu, A.; Tiwari, A. P.; Kim, H. Y., *ACS Applied Energy Materials* **2020,** *3* (4), 3435-3444. DOI 10.1021/acsaem.9b02501.

[28] Liu, T.; Zhang, L.; You, W.; Yu, J., *Small* **2018,** *14* (12), 1702407. DOI https://doi.org/10.1002/smll.201702407.

[29] Dai, S.; Han, F.; Tang, J.; Tang, W., *Electrochimica Acta* **2019,** *328*, 135103. DOI https://doi.org/10.1016/j.electacta.2019.135103.

[30] Wei, G.; Zhou, Z.; Zhao, X.; Zhang, W.; An, C., *ACS Applied Materials & Interfaces* **2018,** *10* (28), 23721-23730. DOI 10.1021/acsami.8b04026.

[31] Jiang, Y.; He, C.; Qiu, S.; Zhang, J.; Wang, X.; Yang, Y., *Chemical Engineering Journal* **2020,** *397*, 125503. DOI https://doi.org/10.1016/j.cej.2020.125503.

[32] Xie, L.; Su, F.; Xie, L.; Li, X.; Liu, Z.; Kong, Q.; Guo, X.; Zhang, Y.; Wan, L.; Li, K.; Lv, C.; Chen, C., *ChemSusChem* **2015,** *8* (17), 2917-2926. DOI https://doi.org/10.1002/cssc.201500355.
[33] Zhang, C.; Xie, L.; Song, W.; Wang, J.; Sun, G.; Li, K., *Journal of Electroanalytical Chemistry* **2013,** *706*, 1-6. DOI https://doi.org/10.1016/j.jelechem.2013.07.032.
[34] Niknam, E.; Naffakh-Moosavy, H.; Moosavifard, S. E.; Afshar, M. G., *Journal of Energy Storage* **2021,** *44*, 103508. DOI https://doi.org/10.1016/j.est.2021.103508.
[35] Zhao, P.; Zhang, Q.; Yang, S.; Chen, L.; Zhu, J., *Journal of Alloys and Compounds* **2021,** *873*, 159725. DOI https://doi.org/10.1016/j.jallcom.2021.159725.
